# The organizing effects of elevated CO_2_ on competition among estuarine primary producers

**DOI:** 10.1038/s41598-017-08178-5

**Published:** 2017-08-09

**Authors:** Craig S. Young, Christopher J. Gobler

**Affiliations:** Stony Brook University, School of Marine and Atmospheric Sciences, Southampton, NY 11968 USA

## Abstract

Fossil fuel combustion, eutrophication, and upwelling introduce excess CO_2_ into coastal zones. The extent to which marine autotrophs may benefit from elevated CO_2_ will be a function of their carbon limitation and, among other factors, competition with other primary producers. Here, we report on experiments performed with North Atlantic species of *Ulva* and *Gracilaria* grown *in situ* or exposed to ambient (~400 µatm) and elevated pCO_2_ (~2500 µatm) and/or subjected to competition with each other and/or with natural plankton assemblages. Elevated pCO_2_ significantly increased the growth rates of *Gracilaria* and *Ulva* and yielded significant declines in tissue δ^13^C, suggesting that increased growth was associated with increased CO_2_ use relative to HCO_3_^−^. *Gracilaria* growth was unaffected by competition with plankton or *Ulva*, while *Ulva* experienced significantly reduced growth when competing with *Gracilaria* or plankton. Dinoflagellates experienced significantly increased growth when exposed to elevated pCO_2_ but significantly slower growth when competing with *Gracilaria*. Elevated carbon-to-nitrogen ratios among macroalgae suggested that competition for nitrogen also shaped interactions among autotrophs, particularly *Ulva*. While some estuarine autotrophs benefit from elevated pCO_2_, the benefit can change when direct competition with other primary producers is considered with *Gracilaria* outcompeting *Ulva* and dinoflagellates outcompeting diatoms under elevated pCO_2_.

## Introduction

By the end of the century, the diffusion of CO_2_ from fossil fuel combustion into surface oceans is expected to cause CO_2_ and HCO_3_^−^ levels to increase 260% and 20%, respectively^1^. Beyond the combustion of fossil fuels, upwelling, and riverine discharge, another prominent CO_2_ source in coastal ecosystems is eutrophication-enhanced microbial respiration^2–4^. The degradation of excessive organic matter can lead to the seasonal accumulation of respiratory CO_2_ which lowers seawater pH and increases pCO_2_ to levels not expected in the open ocean until next century (>1,000 µatm^4^). Shifts in the concentrations of various inorganic carbon sources within the total dissolved inorganic carbon (DIC) pool are likely to elicit a variety of responses from marine flora and fauna. Decreased availability of CO_3_^2−^ can inhibit the growth of calcifying organisms^5–7^, while increased availability of CO_2_ in bulk seawater may benefit some, but not all, photosynthetic organisms^8–11^. The photosynthetic organisms most likely to benefit from an increase in CO_2_ levels are non-calcifying autotrophs whose inorganic carbon uptake is not substrate-saturated at present CO_2_ concentrations^9^, or autotrophs which may gain energetic benefit from the downregulation of processes involved in the actively concentrating carbon internally^12^.

Numerous non-calcified marine autotrophs have been shown to benefit from anthropogenically-induced changes in carbonate chemistry. Marine photosynthetic organisms acquire carbon through the active transport of CO_2_ and HCO_3_^−^ as well as the diffusive uptake of CO_2_^13^. Since HCO_3_^−^ is more abundant than CO_2_ in seawater, many marine autotrophs rely on carbon concentrating mechanisms (CCM) and intracellular or extracellular carbonic anhydrase (CA) to convert HCO_3_^−^ to CO_2_ for use by RuBisCO^9, 13–15^. For marine macroalgae, a variety of chlorophytes, phaeophytes, and rhodophytes are able to utilize HCO_3_^−^ and CO_2_ for photosynthesis^14^. When exposed to elevated CO_2_, some chlorophytes such as *Ulva rigida* and *U. lactuca* experience increased growth^11, 16, 17^, while others do not^18^. Non-calcifying rhodophytes such as *Gracilaria lemaneiformis, G. tikvahiae*, *Chondrus crispus* ^11, 19, 20^, and phaeophytes such as the giant kelp (*Macrocystis pyrifera* ^12^) have been shown to benefit from elevated CO_2_ concentrations. Elevated CO_2_ can also accelerate the growth of individual species of plankton within multiple classes, including dinoflagellates (*Alexandrium fundyense* ^10^, *Karlodinium veneficum* ^21^ *, Alexandrium ostenfeldii* ^22^), diatoms (*Skeletonema costatum* ^23^, *Pseudo-nitzschia multiseries* ^24^ *, Pseudo-nitzschia fraudulenta* ^25^), and raphidophytes (*Heterosigma akashiwo* ^26^). However, not all species within these groups benefit, as is the case of several dinoflagellates^10, 22, 26^. Additionally, some studies have found that natural plankton community growth and composition will be unaffected by increases in pCO_2_ to levels predicted by 2100^27, 28^.

The community structure of marine autotrophs is strongly shaped by competition, which can be affected by relative abundance of resources such as nutrients, light, and inorganic carbon. For example, as nutrient loading increases, macroalgae gain a competitive advantage over seagrass^29^. A similar trend can be found within macroalgal communities, as increased nitrogen loading can favor fast-growing species, such as *Ulva* spp. over slower-growing ones^29^ due to the former possessing higher rates of maximum nutrient uptake^30^. Continued nitrogen loading, however, can shift the competitive advantage in favor of phytoplankton, which often have a higher V_max_, a lower K_m_, and a higher α than macroalgae^31^, thus allowing for faster nutrient acquisition and dominance under conditions of extreme nutrient loading rates and extended residence times^29^. Shifts in the concentration and speciation of inorganic carbon in estuaries may also drive competition among autotrophs. In the presence of high CO_2_, some species of macroalgae may down-regulate their CCMs, thus permitting more energy to be available for other processes such as vegetative growth^9, 11^ or may shift towards diffusive uptake of CO_2_ over use of a CCM to relieve carbon limitation^11, 32^. Some algal species rely strictly on the diffusive uptake of CO_2_ or the active transport of HCO_3_^−^, with most species being capable of using both forms of carbon^14^. Thus, the physiological responses of individual algae to increased CO_2_ may alter community structure^33, 34^.

Recently, we have demonstrated that populations of *Ulva rigida* and *Gracilaria tikvahiae* from Northwest Atlantic coastal waters experience accelerated growth and likely CO_2_ uptake when exposed to elevated pCO_2_^11^. The objective of this study was to assess how elevated concentrations of CO_2_ influences competition among estuarine autotrophs including *Ulva rigida*, *Gracilaria tikvahiae*, diatoms, and dinoflagellates. Each macroalgal population was grown with and without elevated levels of pCO_2_ as well as with and without the other alga, and with and without ambient plankton populations. The growth responses, δ^13^C signatures, and elemental composition of algae were evaluated at the start and end of experiments performed through the growing season of these macroalgal populations.

## Methods

### Macroalgae Collection and Preparation

**Figure 1:**
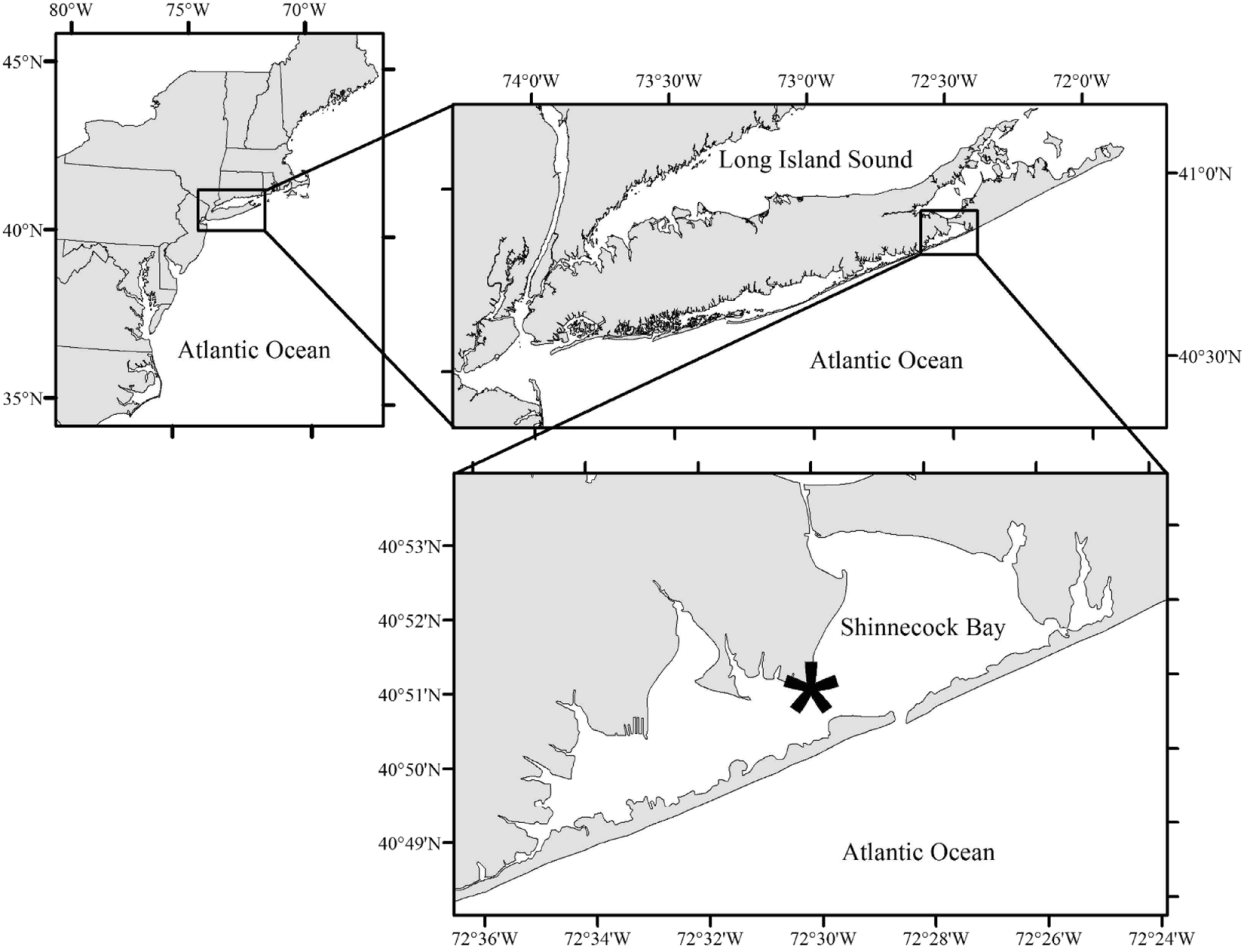
Map of Shinnecock Bay, NY, USA. The asterisk represents the shallow-water region where macroalgal collections occurred and *in situ* experiments were performed. All maps were generated using ArcMap 10.4.1 (Esri).

Macroalgae used for this study were collected from Shinnecock Bay, NY, USA (Fig. 1; 40.85°N, 72.50°) during low tide. Permission to access the water and collect the water and macroalgae was received from the Southampton Town Trustees, Southampton, NY, USA, who hold jurisdiction over Shinnecock Bay. Large, well-pigmented, robust fronds of *Ulva* and *Gracilaria* were collected and transported to the Stony Brook Southampton Marine Science Center in seawater-filled containers within 15 minutes of collection. Prior research has used DNA sequencing and microscopy to determine that *Ulva rigida* and *Gracilaria tikvahiae* are the species of *Ulva* and *Gracilaria* present at the same sampling sites used here during summer and fall^11^. The visual and microscopic analyses during this study affirmed that identification. Due to the plastic nature of macroalgal taxonomic nomenclature as well as the high similarity of ITS sequences among *Ulva* species^35, 36^, for the purposes of this study and consistency with prior studies^11^, we refer to these algae simply as *Ulva* and *Gracilaria*. Individual thalli of *Gracilaria* approximately 5 cm in length were cut from the main plant and placed in a salad spinner to remove debris and epiphytes. Samples were extensively rinsed with filtered (0.2 µm) seawater and placed back into the salad spinner to further remove debris, epiphytes, and excess seawater. Circular sections of similar length of *Ulva* were cut from large thalli with care taken to avoid the outer, potentially reproductive region of the plant^37^. Samples of *Ulva* were prepared using the same cleaning procedures as *Gracilaria*. All samples were weighed on an A&D EJ300 digital balance (±0.01 g) to obtain initial wet weight in grams. To prevent desiccation, all samples were kept in 100 mL filtered (0.2 µm) seawater-filled containers after spinning and weighing but prior to use in experiments.

### *In situ* Growth Experiments

To assess growth rates of *Gracilaria* and *Ulva* within the region of Shinnecock Bay from which they were collected, *in situ* growth experiments were performed monthly from June through October. Quadruplet, 0.25 m^2^ incubation cages constructed from 1 cm^2^ wire mesh were attached to a four-armed (25 cm) umbrella fishing apparatus on a line with surface flotation and a bottom weight to keep the cages suspended at 0.2 m^11, 37^. Continuous measurements of light and temperature were made using HOBO pendant temperature and light loggers. Thalli of each species of macroalgae were placed in each quadruplet cage for approximately one week in parallel with laboratory experiments (*described below*) after which thalli were recovered, brought to the lab, and rinsed, spun, re-rinsed, re-spun, and weighed as described above. Samples of *Gracilaria* and *Ulva* were frozen for further tissue analysis. Weight-based growth rates for both species were determined using the relative growth rate formula (growth d^−1^) = (ln W_final_ − ln W_initial_)/(Δt), where W_final_ and W_initial_ are the final and initial weights in grams and Δt is the number of days of the experiment.

### Assessing the Effects of Elevated pCO_2_ and Competition

Five laboratory experiments were performed to assess the effects of competition and elevated pCO_2_ on the growth of *Gracilaria, Ulva*, and natural plankton communities during early July, late July, August, September and October. Polycarbonate bottles (2.5 L) were acid washed (10% HCl) and liberally rinsed with deionized water before use. Experimental bottles were placed in an environmental control chamber set to the approximate temperature (~16–21 °C) and light intensity (~400 µmol s^−1^ m^−2^) and duration (14 h: 10 h light: dark cycle) present during *in situ* experiments. Bottles were filled with filtered (0.2µm polysulfone filter capsule, Pall) with the plankton community removed or unfiltered seawater with the full plankton community. For the early and late July, and August experiments, bottles were randomly assigned and dispersed, in triplicate, to one of four treatments: a control with ambient levels of pCO_2_ (~400 µatm) in filtered seawater (no plankton), a treatment with ambient pCO_2_ in unfiltered seawater (with plankton), a treatment with elevated pCO_2_ (~2,500 µatm) in filtered seawater (no plankton), and a treatment with elevated pCO_2_ in unfiltered seawater (with plankton). Three sets of these bottles were established: One for *Ulva*, one for *Gracilaria*, and one with both *Ulva* and *Gracilaria* resulting in a total of 36 experimental bottles. For the September and October experiments, bottles were randomly assigned and dispersed to the aforementioned treatments, but in quadruplicate. Additionally, eight bottles were filled with seawater only with four bottles being subjected to ambient pCO_2_, and the other four being subjected to elevated pCO_2_. All bottles for each experiment received nutrient additions (50µM nitrate, 3 µM phosphate) at the beginning of the experiment to ensure nutrient replete growth. The nutrient and pCO_2_ concentrations used during experiments were higher than what is present at the collection site, but are within the range of concentrations present in eutrophic US East Coast estuaries^4, 37^ and used during prior experiments with *Ulva* and *Gracilaria* from Shinnecock Bay, NY, USA^11^.

Each bottle was aerated via 3.8 × 1.3 cm air diffusers (Pentair) connected to a 1 mL, polystyrene serological pipette inserted to the bottom of each bottle and connected via tygon tubing to an air source. Bottles were subjected to the control (~400 µatm) and elevated (~2500 µatm) levels of pCO_2_ via a gas proportionator system (Cole Parmer® Flowmeter system, multitube frame) that mixed ambient air with 5% CO_2_ gas (δ^13^C = −28‰)^5^. The gas mixtures were delivered at a net flow rate of 2500 ± 5 mL min^−1^ through an 18- or 14-way gang valve into the serological pipettes that fit through an opening in the closed cap of the bottle. The delivery rate of gases turned over the volume of the experimental bottles >1,000 times daily^5^ and bottles were left uncapped but covered with aluminum foil to permit gas exchange Bubbling began two days prior to beginning each experiment allowing pCO_2_ concentrations and pH levels to reach a state of equilibrium. Experiments persisted for one week. Measurements of pH within bottles were made daily through use of an Orion Star A321 Plus electrode (±0.001) calibrated prior to use with National Institute of Standards and Technology (NIST) traceable standards. DIC concentrations in bottles were measured using an EGM-4 Environmental Gas Analyzer (PP Systems) system that quantifies DIC levels after separating the gas phase from seawater by acidification using a Liqui-Cel Membrane (Membrana)^5^. As a quality assurance measure, the levels of DIC and pH with Dr. Andrew Dickson’s (University of California, San Diego, Scripps Institution of Oceanography) certified reference material (Batches 142, 147, 151 = 2038, 2014, and 2033 µmol DIC kg seawater^−1^, respectively) were measured during analyses of every set of samples. The analysis of samples continued only after complete recovery of the certified reference material was attained. The measured values were 104 ± 3.9% of the certified values. Levels of pCO_2_ (mean of t = initial and t = final, Table 1) were calculated using measured levels of DIC, pH (NIST), temperature, and salinity, as well as the first and second dissociation constants of carbonic acid in seawater^38^ using the program CO2SYS (http://cdiac.ornl.gov/ftp/co2sys/). The targeted levels of pCO_2_ resulted in actual pCO_2_ and pH values of ~400 µatm and ~8.0, respectively, for ambient conditions and ~2600 µatm and ~7.2, respectively, for the elevated CO_2_ conditions, mimicking the range found seasonally in estuarine environments^3, 4, 39^.

**Table 1:** Values of pH (NBS scale), temperature (°C), salinity (g kg^−1^), pCO_2_ (µatm), DIC (µmol kgSW^−1^), HCO_3_^−^ (µmol kgSW^−1^) for *Gracilaria* and *Ulva* for June through October experiments.

*Ulva*						
Treatment	pH	Salinity	Temperature	pCO_2_	DIC	HCO_3_^−^
Ambient/Filtered	8.14 ± 0.04	30.9 ± 0.5	16.6 ± 0.5	270 ± 30	1230 ± 30	1140 ± 30
Ambient/Unfiltered	8.23 ± 0.04	30.7 ± 0.6	16.5 ± 0.6	270 ± 30	1490 ± 60	1360 ± 50
CO_2_/Filtered	7.17 ± 0.04	30.3 ± 0.3	15.7 ± 0.5	2600 ± 200	1490 ± 60	1370 ± 50
CO_2_/Unfiltered	7.26 ± 0.04	30.8 ± 0.5	15.9 ± 0.7	2660 ± 240	1630 ± 50	1520 ± 40
***Gracilaria***						
Ambient/Filtered	8.10 ± 0.04	30.9 ± 0.5	16.0 ± 0.7	300 ± 30	1280 ± 30	1190 ± 30
Ambient/Unfiltered	8.19 ± 0.05	30.7 ± 0.6	16.5 ± 0.6	310 ± 40	1630 ± 100	1490 ± 90
CO_2_/Filtered	7.17 ± 0.04	30.4 ± 0.4	15.0 ± 0.6	2670 ± 260	1450 ± 60	1330 ± 50
CO_2_/Unfiltered	7.26 ± 0.4	30.7 ± 0.6	15.5 ± 0.5	2550 ± 250	1670 ± 60	1550 ± 50
***Gracilaria*** **and** ***Ulva***						
Ambient/Filtered	8.15 ± 0.04	30.9 ± 0.5	16.4 ± 0.7	270 ± 20	1240 ± 30	1150 ± 30
Ambient/Unfiltered	8.22 ± 0.06	30.6 ± 0.5	16.3 ± 0.5	280 ± 40	1540 ± 40	1410 ± 30
CO_2_/Filtered	7.16 ± 0.04	30.5 ± 0.4	15.6 ± 0.5	2520 ± 180	1450 ± 50	1320 ± 50
CO_2_/Unfiltered	7.27 ± 0.04	30.6 ± 0.5	15.8 ± 0.5	2700 ± 230	1660 ± 50	1550 ± 50

Values represent means ± standard error. Data from individual experiments appear within supplementary Tables (S1 Table).

Experiments began with the introduction of macroalgae and nutrients into experimental bottles. HOBO pendant temperature and light data loggers were used to continuously monitor light levels. At the end of experiments, final pH, temperature, and salinity measurements were made and a final DIC was collected and analyzed as described above. After measuring DIC, all macroalgae samples were removed from their respective bottles and rinsed, spun, re-rinsed, re-spun, and weighed as described above. *Gracilaria* and *Ulva* samples were placed into small freezer bags for further analyses. Weight-based growth rates for both species were determined as described above. Significant differences in growth rates were assessed using three-way ANOVA with SigmaPlot 11.0, where the main treatments were pCO_2_ treatment (ambient or elevated), the presence of plankton (filtered or unfiltered seawater), and competition (each macroalgal species alone or in the same bottle). Additionally, one-way ANOVA were used to compare the growth rates of the control group and the *in situ* experiments.

The growth and composition of the plankton community was assessed during the September and October experiments by removing 50 mL aliquots of seawater from experimental bottles in unfiltered seawater treatments at the beginning and at the conclusion of each experiment and preserving samples with Lugol’s iodine. Aliquots were placed in Sedgewick-Rafter chambers and enumerated using a light microscope, an approach that permitted the quantification of plankton >10 µm^37^. More than 200 cells were quantified per sample. For the purposes of this study, the most abundant phytoplankton groups were quantified, specifically diatoms and dinoflagellates. Significant differences in abundance were assessed using three-way ANOVA with SigmaPlot 11 where the main treatments were pCO_2_ (ambient or elevated), *Ulva* (with or without *Ulva*), and *Gracilaria* (with or without *Gracilaria*).

### Tissue Analyses

For carbon (C), nitrogen (N), and stable carbon isotope (δ^13^C) analyses, frozen samples of *Gracilaria* and *Ulva* were dried at 55 °C for 48 h and then homogenized into a fine powder using a mortar and pestle. The total tissue C, N, and δ^13^C were analyzed using an elemental analyzer interfaced to a Europa 20–20 isotope ratio mass spectrometer at the UC Davis Stable Isotope Facility. Significant differences in tissue content for each species of algae and class of phytoplankton during experiments were assessed using three-way ANOVA within SigmaPlot 11.0 where the main treatment effects were pCO_2_ treatment (ambient or elevated), the presence of plankton (filtered or unfiltered seawater), and competition (each macroalgal species alone or in the same bottle).

Lastly, we made use of an isotopic mixing model to estimate the use of CO_2_ and HCO_3_^−^ during experiments^11^. This model considered the δ^13^C and biomass of macroalgal tissue before and after experiments, the δ^13^C of the 5% CO_2_ gas used for the experiments (−28‰), the δ^13^C of the marine CO_2_ and HCO_3_^−^ pool (−10‰ and 0‰, respectively^40–42^), C fractionation during macroalgal uptake of CO_2_ and HCO_3_^−^ (−20‰ and −10‰, respectively^40–42^), C fractionation during conversion of the 5% CO_2_ gas bubbled into the experimental containers to HCO_3_^−^ (+10‰)^40–42^, and the DIC concentration with and without the addition of the 5% CO_2_ gas. The latter provides indication of the fraction of DIC contributed by the tanked CO_2_ gas compared to ambient air. The model assumed that the tanked CO_2_ reached equilibrium with the total DIC pool, allowing the HCO_3_^−^ pool to assume a lighter δ^13^C signature proportional to the fraction of the DIC pool comprised of tanked CO_2_ compared to ambient air, an assumption supported by the high turnover rate of seawater by the bubbled CO_2_ mixture (1000-times daily). Due to the macroalgal tissue being dried and homogenized, it was assumed that the δ^13^C signature of the macroalgal tissue was representative of the fraction of original tissue with its original δ^13^C and the tissue grown during the experiment taking on a δ^13^C signature representative of the DIC pool with a value made proportionally more negative (lighter) by the tanked CO_2_ gas^11^. Finally, two sets of mixing models were run for each macroalgal species that estimated their δ^13^C signature based on exclusively CO_2_ and exclusively HCO_3_^−^ during the experiments^11^. A one-way ANOVA was used to assess the differences between the measured δ^13^C signatures of the macroalgae and signatures calculated based on exclusive use of either CO_2_ or HCO_3_^−^, with Tukey tests used to assess the differences between individual groups.

## Results

### *Gracilaria*

**Figure 2:**
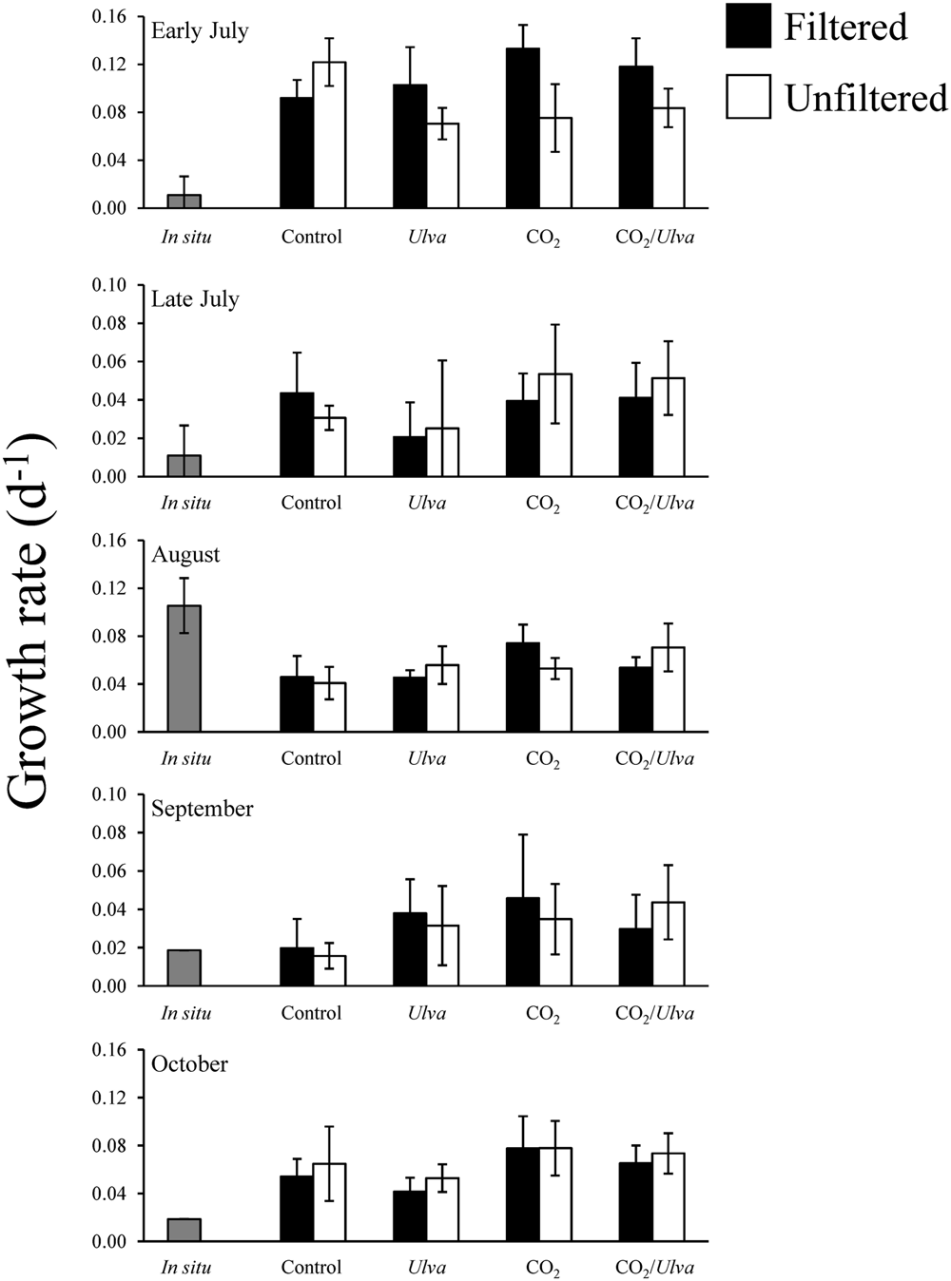
Growth rates of *Gracilaria* exposed to ambient and elevated CO_2_ conditions, with and without competition from *Ulva*, and with and without competition from plankton for experiments performed July through October. For three-way ANOVA, CO_2_ was a main treatment effect during the late July, August, and October experiments. The presence of plankton was a main treatment effect during the early July experiment (see Supplementary Table S2).

The *in situ* growth of *Gracilaria* in Shinnecock Bay was found to be similar to and not significantly different from growth rates within the control groups of experiments, with the exception of the early July and August experiment, when experimental growth rates were slightly lower and higher, respectively, than those *in situ* (One-way ANOVA; *p* < 0.05; Fig. 2; Supplementary Table S2). The growth rates of *Gracilaria* within the experimental groups were found to be sensitive to changes in CO_2_ concentrations (Fig. 2). During experiments in late July, August, and October, the growth of *Gracilaria* increased significantly when exposed to elevated CO_2_ concentrations (Three-way ANOVA; *p* < 0.05; Fig. 2; Supplementary Table S2). On average, growth rates under elevated CO_2_ were 37% higher and 30% higher than growth under ambient conditions in experimental bottles filled with filtered and unfiltered seawater, respectively (Fig. 2). Growth rates of *Gracilaria* were not affected by the presence of *Ulva* and were mostly unaffected by the presence of plankton with the exception of the early July experiment when plankton significantly slowed the growth of *Gracilaria* (Three-way ANOVA; *p* < 0.05; Fig. 2; Supplementary Table S2). During the August experiment, there was an interaction between CO_2_, competition with *Ulva*, and competition with plankton, whereby elevated CO_2_ significantly enhanced growth rates within filtered treatments (Three-way ANOVA; *p* < 0.05; Fig. 2; Supplementary Table S2) but not within unfiltered treatments (Three-way ANOVA; *p* > 0.05; Fig. 2; Supplementary Table S2). Additionally, in this same experiment, growth was significantly higher under elevated CO_2_ in treatments without *Ulva* (Three-way ANOVA; *p* < 0.05; Fig. 2; Supplementary Table S2), but not in treatments with competition from *Ulva*, demonstrating that *Ulva* altered the response of *Gracilaria* to CO_2_ in this experiment.

**Figure 3:**
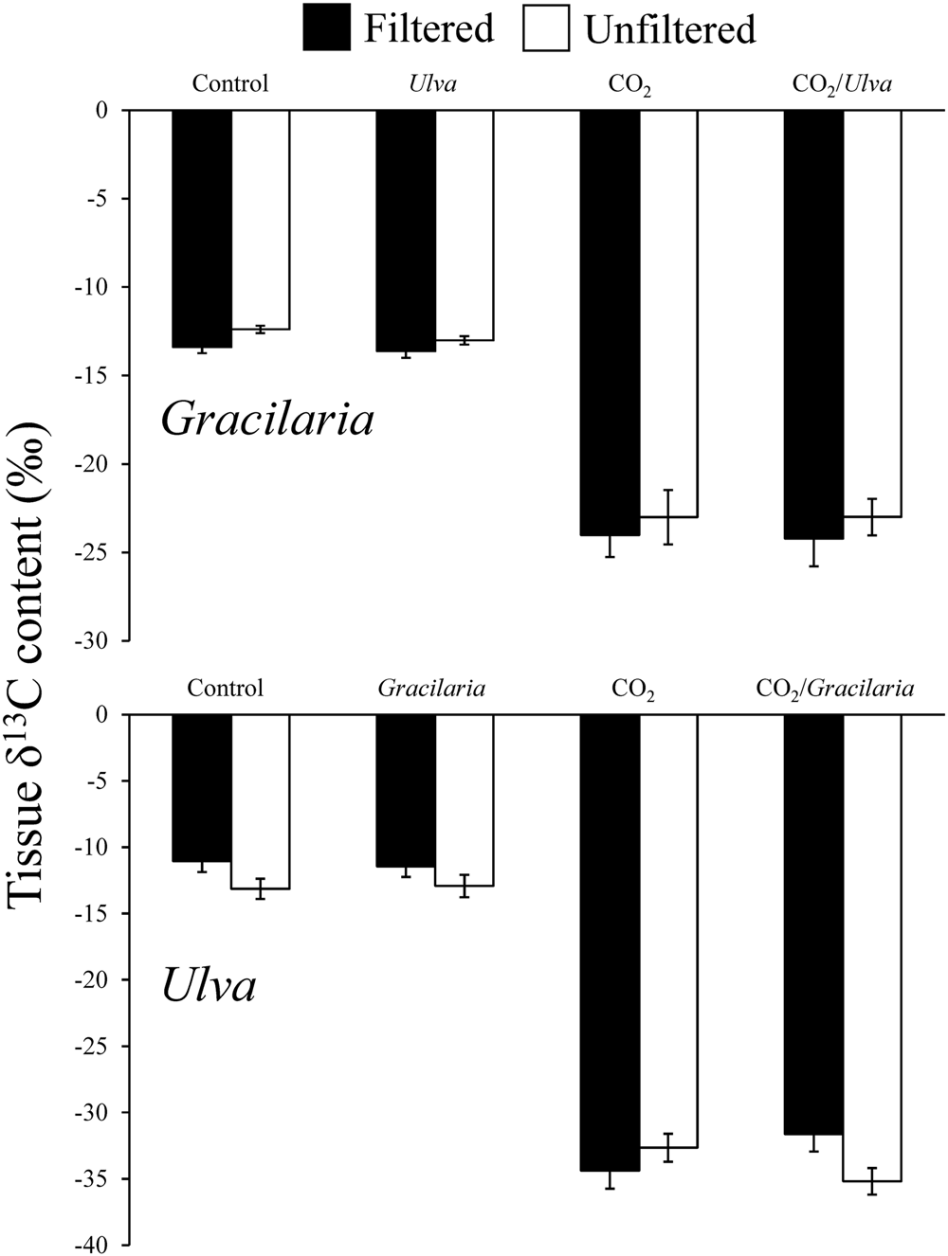
δ^13^C content of *Gracilaria* and *Ulva* exposed to ambient and elevated CO_2_ conditions, with and without competition from *Ulva*, and with and without competition from plankton for experiments performed July through October. For three-way ANOVA, CO_2_ was a main treatment effect, on average (see Supplementary Table S2).

**Figure 4:**
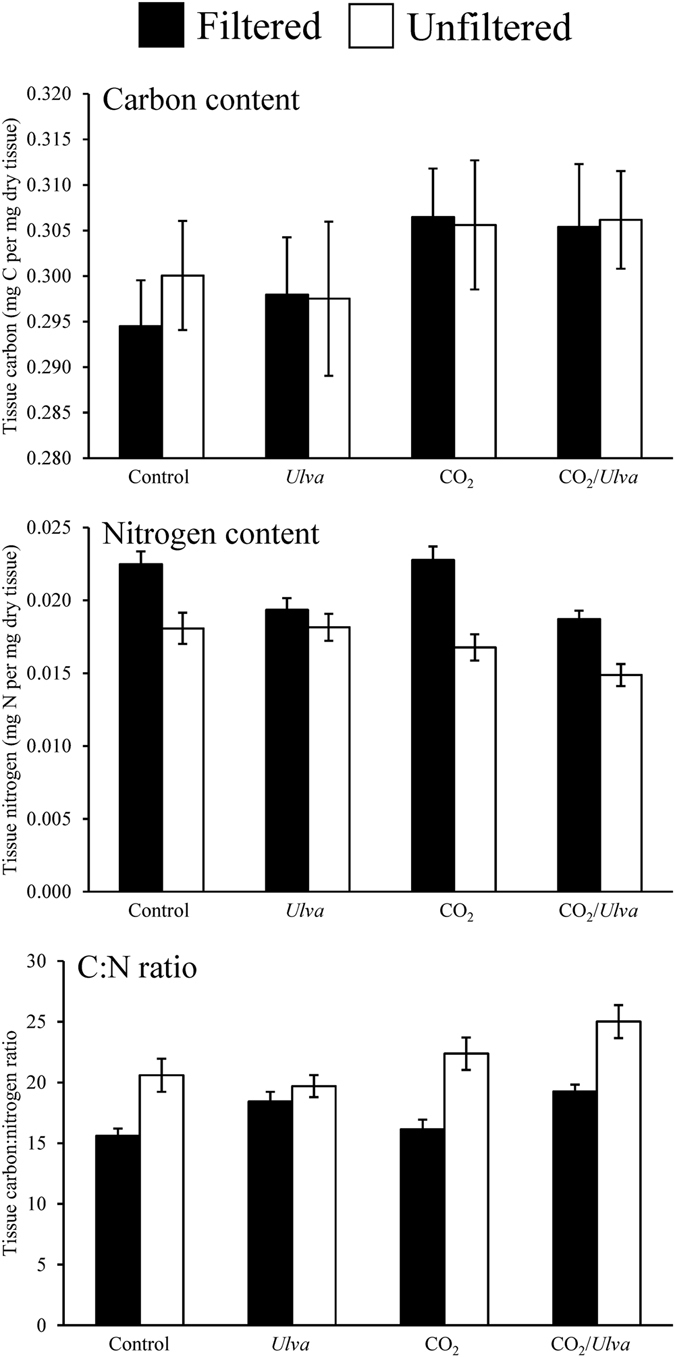
Tissue nitrogen, carbon, and C:N content of *Gracilaria* exposed to ambient and elevated CO_2_ conditions, with and without competition from *Ulva*, and with and without competition from plankton for experiments performed July through October. For tissue N and C:N ratio, CO_2_, the presence of plankton (un/filtered), and the presence of *Ulva* were main treatment effects of three-way ANOVA, on average (see Supplementary Table S2).

The δ^13^C content of *Gracilaria* was significantly reduced by elevated CO_2_ delivery, with the average of the ambient and elevated CO_2_ treatments being, −13‰ and −24‰, respectively (Three-way ANOVA; *p* < 0.001; Fig. 3; Supplementary Tables S2-S3). Overall, there was no significant difference in δ^13^C between filtered and unfiltered seawater treatments, regardless of CO_2_ concentration (Three-way ANOVA; *p* > 0.05; Supplementary Tables S2-S3). Additionally, there was no significant difference in δ^13^C caused by exposure to *Ulva*. Isotopic mixing models demonstrated that, when exposed to elevated CO_2_ concentrations, the δ^13^C signatures of *Gracilaria* (−24‰) were significantly lower than values expected if their C was obtained exclusively from use of HCO_3_^−^ (−14‰; Tukey test; *p* < 0.05; Supplementary Fig. S1), but not significant different than expected from exclusive use of CO_2_ (−28‰; Tukey test; *p* > 0.05; Supplementary Fig. S1). On average, the tissue C content of *Gracilaria* was largely unaffected by CO_2_ concentration, competition with *Ulva*, and competition with plankton (Three-way ANOVA; *p* > 0.05; Fig. 4; Supplementary Tables S2 and S4). However, elevated CO_2_ was found to have significantly increased the tissue C content relative to the ambient concentration for the late July experiment (Three-way ANOVA; *p* < 0.05; Supplementary Table S2). Competition with *Ulva* significantly reduced tissue N of *Gracilaria* for the August, September, and October experiments, while competition with plankton significantly decreased tissue N for all experiments with the exception of the August experiment (Three-way ANOVA; *p* < 0.05; Supplementary Tables S2 and S4). Elevated CO_2_ treatments resulted in decreased tissue N for only the September experiment (S2 and S4 Tables). The tissue C:N ratio of *Gracilaria* was unaffected by elevated CO_2_ concentrations (Three-way ANOVA; *p* > 0.05; Supplementary Tables S2 and S4), but was found to be significantly higher during competition with *Ulva* during the August experiment and during competition plankton assemblages during the early and late July experiments (Three-way ANOVA; *p* < 0.05; Fig. 4; Supplementary Tables S2 and S4).

**Figure 5:**
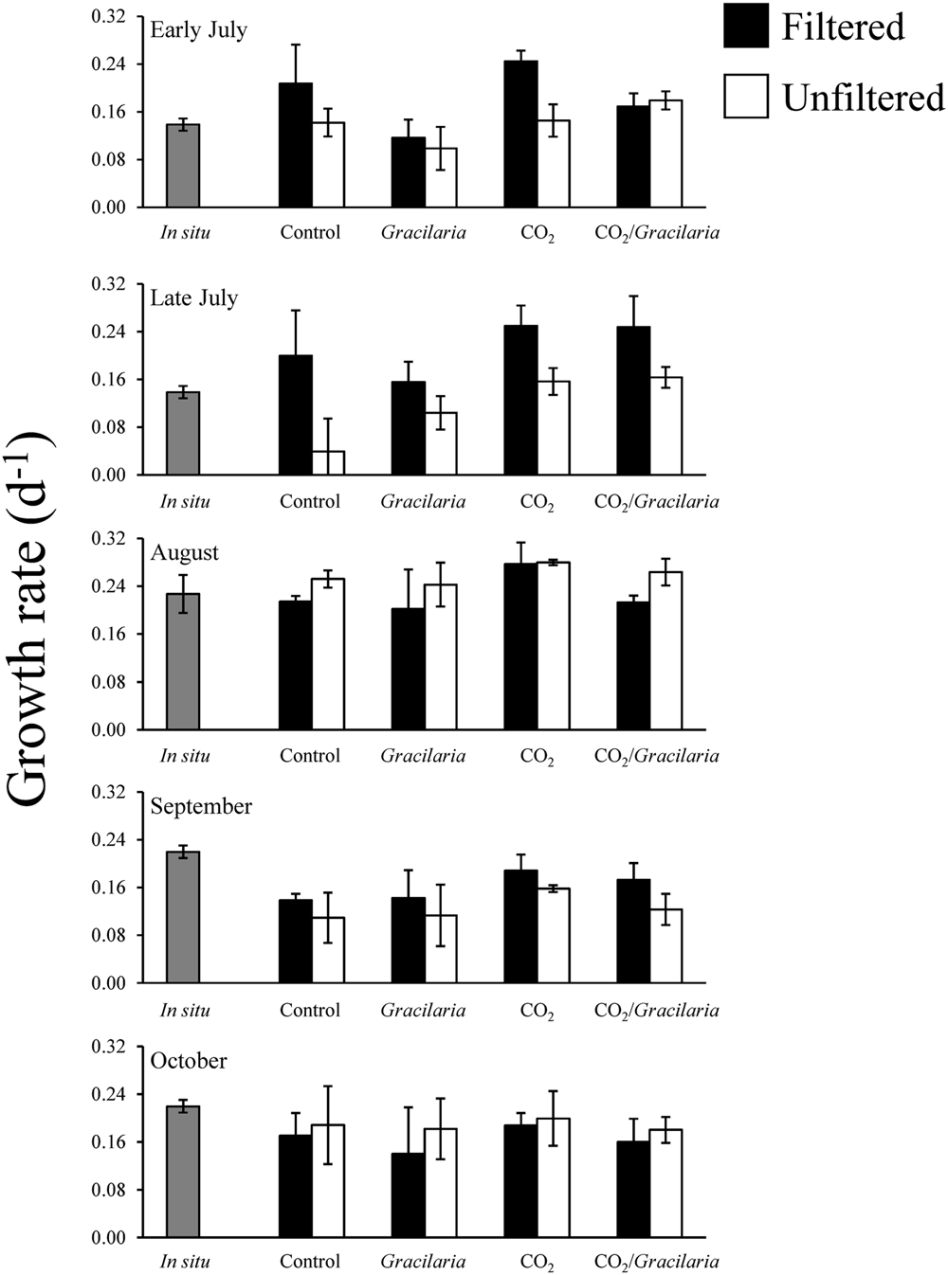
Growth rates of *Ulva* exposed to ambient and elevated CO_2_ conditions, with and without competition from *Gracilaria*, and with and without competition from plankton for experiments performed July through October. For three-way ANOVA, CO_2_ was a main treatment effect during the early and late July, August, and September experiments. The presence of plankton was a main treatment effect during the early and late July experiments. The presence of *Gracilaria* was a main treatment effect during the early July and August experiments (see Supplementary Table S2).

### *Ulva*

The growth rates of *Ulva* during *in situ* experiments did not differ statistically from those found within the control treatment of experiments (One-way ANOVA; *p* > 0.05; Fig. 5; Supplementary Table S2). The response of *Ulva* to the different variables within the experimental bottles was more complex compared to *Gracilaria*. Overall, growth by *Ulva* was significantly higher under elevated pCO_2_ concentrations and significantly higher in treatments without *Gracilaria* and competing plankton (Three-way ANOVA; *p* < 0.05; Fig. 5; Supplementary Table S2). During four of the five experiments (early and late July, August, and September), the growth of *Ulva* increased significantly when exposed to elevated pCO_2_ concentration, increasing, on average, 38% and 44% relative to ambient treatments in filtered and unfiltered treatments, respectively (Three-way ANOVA; *p* < 0.05; Fig. 5; Supplementary Table S2). On average, *Ulva* growth rates were ~20% lower when grown in the presence of plankton, and 12% lower when grown in the presence of *Gracilaria* (Fig. 5). During the early July experiment, the presence of plankton depressed the growth of *Ulva* as did the presence of *Gracilaria* (Three-way ANOVA; *p* < 0.05; Fig. 5; Supplementary Table S2). *Ulva* growth in the presence of plankton was also significantly reduced during the late July experiment (Three-way ANOVA; *p* < 0.05; Fig. 5; Supplementary Table S2). As independent variables, plankton and *Gracilaria* did not significantly alter *Ulva* growth rates during the September experiment, but there was a synergistic interaction between elevated pCO_2_ and the absence of plankton in slowing *Ulva* growth (Three-way ANOVA; *p* < 0.05; Fig. 5; Supplementary Table S2). During the October experiment, the growth of *Ulva* was not affected by any treatment.

**Figure 6:**
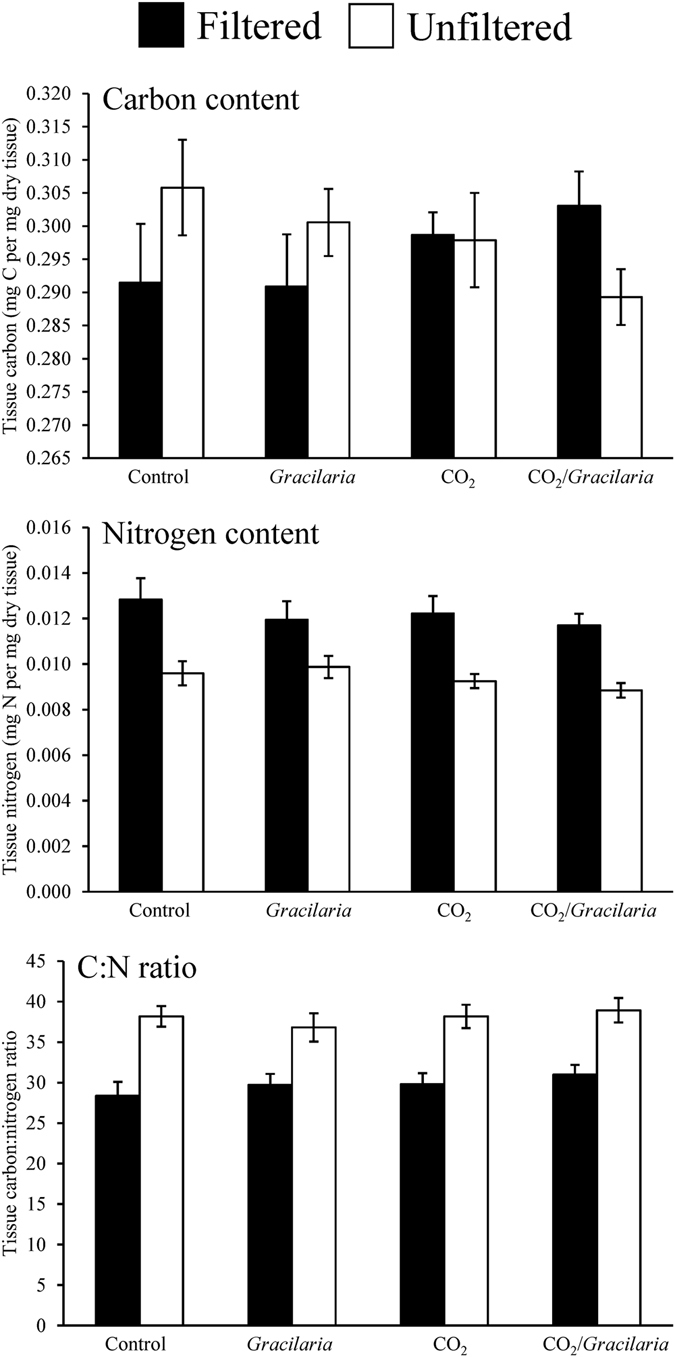
Tissue nitrogen, carbon, and C:N content of *Ulva* exposed to ambient and elevated CO_2_ conditions, with and without competition from *Gracilaria*, and with and without competition from plankton for experiments performed July through October. For tissue N and C:N ratio the presence of plankton was a main treatment effect of three-way ANOVA, on average (see Supplementary Table S2).

The δ^13^C content of *Ulva* was significantly reduced by exposure to elevated CO_2_ concentrations, with the average δ^13^C of the ambient and elevated CO_2_ treatments being −12‰ and −33‰, respectively (Three-way ANOVA; *p* < 0.001; Fig. 3; Supplementary Tables S2-S3). For the entire study, the δ^13^C of *Ulva* was not significantly altered by the presence of *Gracilaria* or plankton (Three-way ANOVA; *p* > 0.05; Supplementary Tables S2-S3). The δ^13^C was, however, found to be significantly lower in treatments with plankton present for the August and September experiments (Three-way ANOVA; *p* < 0.05; Supplementary Tables S2-S3). Isotopic mixing models demonstrated that when exposed to elevated CO_2_ concentrations, *Ulva* δ^13^C signatures (−33‰) were significantly lower than values expected from exclusive use of HCO_3_^−^ (−14‰; Tukey test; *p* < 0.05; Supplementary Fig. S1) and significantly higher than expected from exclusive use of CO_2_ (−45‰; Tukey test; *p* < 0.05; Supplementary Fig. S1). Tissue C content of *Ulva* was not significantly affected by elevated CO_2_ concentrations, competition with *Gracilaria*, or competition with plankton (Three-way ANOVA; *p* < 0.05; Fig. 6; Supplementary Tables S2 and S4). In contrast, during each experiment tissue N content was significantly lower when *Ulva* was grown in the presence of plankton, with the exception of the October experiment (Three-way ANOVA; *p* < 0.05; Fig. 6; Supplementary Tables S2 and S4). The tissue C:N ratio of *Ulva* was significantly higher in the presence of plankton during each experiment except October (Three-way ANOVA; *p* < 0.05; Fig. 6; Supplementary Tables S2 and S4).

**Figure 7:**
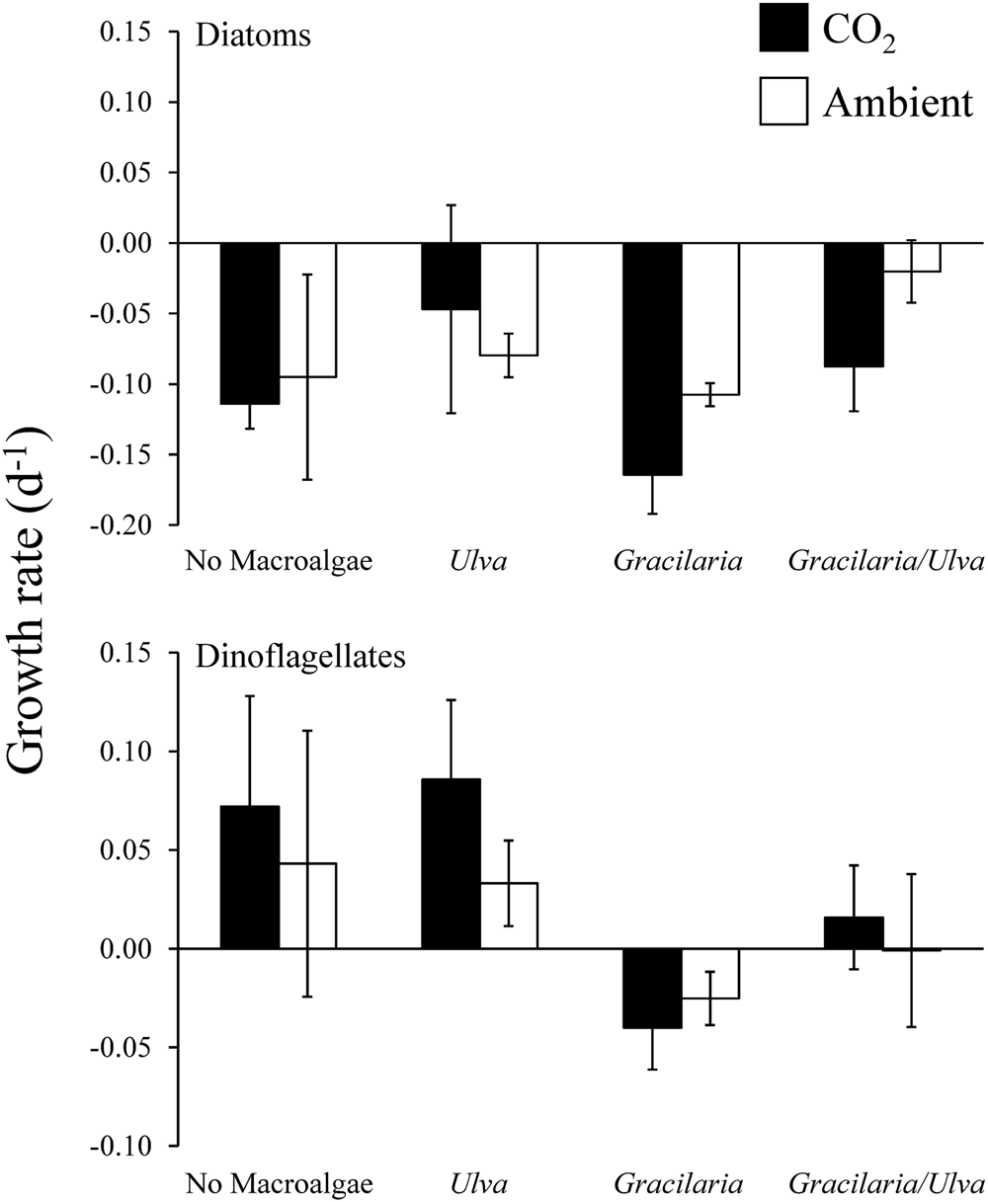
Growth rates of diatoms and dinoflagellates exposed to ambient and elevated CO_2_ conditions, with and without competition from *Gracilaria* and/or *Ulva*. On average, the presence of *Ulva* and *Gracilaria* were main treatment effects of three-way ANOVA for diatoms and dinoflagellates, respectively (see Supplementary Table S2).

### Phytoplankton

Regarding phytoplankton communities, at the onset of the September and October experiments, the dominant phytoplankton >10 µm were diatoms, whereas at the end of experiments, the abundance of diatoms diminished and dinoflagellates became more prominent. The growth rates of diatoms and dinoflagellates were found to significantly decrease and increase, respectively, during exposure to elevated CO_2_ during the September and October experiments (Three-way ANOVA; *p* < 0.05; Fig. 7; Supplementary Table S2). Diatoms and dinoflagellate growth rates were also affected by the species of macroalgae present. Diatom growth rates were significantly higher in treatments containing *Ulva* compared to treatments without (Three-way ANOVA; *p* < 0.05; Fig. 7; Supplementary Table S2). Dinoflagellates growth was significantly decreased in the presence of *Gracilaria* (Three-way ANOVA; *p* < 0.05; Fig. 7; Supplementary Table S2).

**Figure 8:**
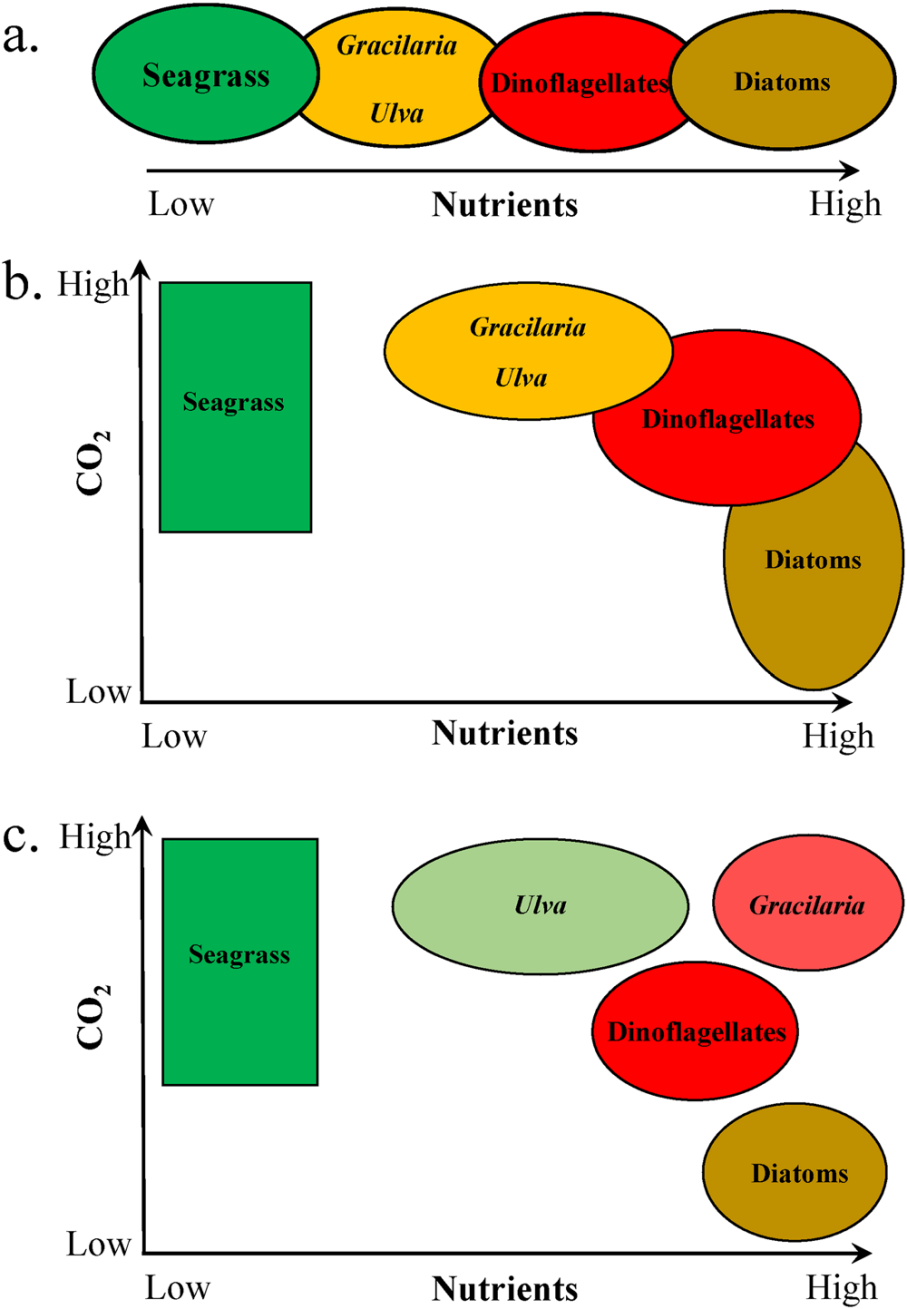
Responses and interactions of various estuarine primary producers to eutrophication, ocean acidification, and competition under three scenarios: (**a**) Nutrient loading only, with competition. (**b**) Low to elevated CO_2_ and nutrient loading, without competition. (**c**) Low to elevated CO_2_ and nutrient loading, with competition.

## Discussion

During this study, elevated CO_2_ concentrations significantly enhanced the growth rates of *Gracilaria*, *Ulva*, and dinoflagellates, but not diatoms. For *Gracilaria*, growth rates were largely unaffected by the presence of *Ulva* and plankton whereas the growth rates of *Ulva* were significantly depressed when grown with *Gracilaria* or the full plankton community. Among the phytoplankton, diatom growth benefited from the presence of *Ulva*, while the growth rates of dinoflagellates were slowed by *Gracilaria*. For both macroalgae, tissue δ^13^C was significantly lowered by elevated pCO_2_ while tissue N content was reduced by competition with the other macroalgae species and/or plankton. While these experiments were performed within bottles, the rapid turnover of the dissolved gas pools yielded growth rates of macroalgae that were nearly identical to parallel thalli concurrently measured in an ecosystem setting evidencing the realistic nature of conditions during experiments. Collectively, these findings provide novel insight regarding the outcomes of competition among primary producers under high CO_2_ conditions.

Most macroalgae are capable of active transport of HCO_3_^−^ or CO_2_ into their CCM or the diffusive uptake of CO_2_^13^. High CO_2_ concentrations may cause macroalgae to down-regulate CCMs that convert HCO_3_^−^ to CO_2_^16, 19, 43, 44^ resulting in more energy available for other processes such as vegetative growth^9, 11^. The amount of energy saved by this process is not fully clear, as the process depends on several external factors, such as PAR, and internal factors, such as type of CCM used by the macroalgae, or the potential leakage of carbon dioxide from the CCM^45^. The δ^13^C signatures of macroalgae during this study suggested these species switched from mostly HCO_3_^−^ to more CO_2_ use and potentially downregulated their CCMs. Values prior to the start of the experiments (−12–13‰) were reflective of HCO_3_^−^ and CCM use whereas the more negative values of macroalgae at the end of the experiment (−23.6 ± 5‰ and −33.5 ± 5‰ for *Gracilaria* and *Ulva*, respectively) were within the range expected of macroalgae relying more on the diffusive uptake of CO_2_^12, 40, 46^ using isotope mixing models to account for the lighter CO_2_ gas used in experiments^11^. It is also possible that higher pCO_2_ alleviated inorganic C limitation and enhanced growth rates. Mercado *et al*.^32^ reported that *U. rigida* and *U. compressa* (formerly *Enteromorpha*) do not receive enough CO_2_ through diffusive uptake at current CO_2_ levels, a finding consistent with the enhanced growth of *Ulva* during this study and supported by the shift in δ^13^C during this study for both *Ulva* and *Gracilaria*. Regardless, the enhanced growth rates for these macroalgae under higher CO_2_ indicate that inorganic C limitation was alleviated.

Consistent with prior studies of macroalgae, changes in CO_2_ levels did not alter tissue C and N content^11, 47^ and competition with other autotrophs did not alter their C content. In contrast, competition with other autotrophs resulted in significantly decreased N content and decreased tissue C:N ratios for *Gracilaria* and *Ulva*. Both macroalgal species are able to rapidly assimilate and store nitrate^48, 49^ and have been shown to experience enhanced tissue N content when exposed to excessive nitrate concentrations^50, 51^. Compared to *Gracilaria*, *Ulva* is capable of undergoing more rapid growth in eutrophic settings^29, 37^ due to a high maximum rate of uptake of nutrients such as nitrate^30^. Phytoplankton are superior competitors for N compared to macroalgae^29, 31^. The significant declines in N content of macroalgae when grown with plankton and elevated C:N ratios of macroalgae at the end of experiments (15–40), despite the high levels of N present at the start of experiments (50 µM), affirms the role of N as a limiting element in this^52^ and other estuaries^53^, and suggests this N was likely depleted over the course of the experiment. This is almost certainly the case in experiments with the full plankton community intact as uptake rates of plankton communities can exceed 25 µmol L^−1^ day^−1^ in Shinnecock Bay^52^. The precise outcomes of competition among estuarine autotrophs exposed to high CO_2_, therefore, will be partly dependent upon ambient nutrient supplies.

Beyond tissue content of macroalgae, the importance of both N and pCO_2_ in shaping algal community composition was also evident in the competitive growth responses of macroalgae. The ability of macroalgae to respond to shifts in the ratio of HCO_3_^−^ to CO_2_ in seawater may prompt algae capable of using both inorganic carbon species to gain a competitive advantage over algae restricted to only HCO_3_^−^. Cornwall *et al*.^34^ found that macroalgal abundance along a CO_2_ gradient at Vulcano, Italty varied according to the inorganic carbon uptake strategy of the algae. During that study, five macroalgae species capable of using HCO_3_^−^ and CO_2_ increased with abundance as CO_2_ concentrations increased, as well showed a decline in tissue δ^13^C associated with increased CO_2_ use. However, calcifying macroalgae, as well as species incapable of using CO_2_, decreased with abundance as CO_2_ concentrations increased^34^. During the present study, *Ulva* and *Gracilaria*, when exposed to elevated CO_2_, had increased growth and declines in tissue δ^13^C associated with increased CO_2_ use, which indicates that both may gain a competitive advantage over species incapable of adjusting their inorganic carbon physiology in response to increases in CO_2_ in seawater. But besides carbon use strategies, competition for nutrients is also a key factor to consider. Nutrient loading favors fast-growing macroalgae with rapid uptake rates of nutrients over slower-growing counterparts^29, 30^. The growth rates of *Ulva* were, on average, three-times faster than *Gracilaria* during experiments and thus, despite a 55–60% lower tissue N content, had a significantly larger N demand, making *Ulva* more prone to N limitation, especially when placed in competition with other autotrophs. This hypothesis is supported by the C:N ratios of *Ulva* which were significantly higher than those of *Gracilaria* throughout this study (*p* < 0.001; T-test), suggesting *Ulva* was more N-limited. Similarly, the presence of plankton, which are able to outcompete macroalgae for nutrients, may have further depleted nutrient concentrations during experiments, thus causing the decreased growth of *Ulva* in unfiltered treatments for some of the experiments^29^. Again, this hypothesis is supported by the significant increase in the C:N ratio that *Ulva* experienced when grown in the presence of plankton communities. Collectively these findings suggest that while *p*CO_2_ enhances the growth of *Ulva* and *Gracilaria*, the slower-growing *Gracilaria* is better adapted for persisting at more dynamic nutrient concentrations than *Ulva* ^30, 54^. In a field experiment by Fujita (1985)^55^, when N was introduced in pulses every five days, *Gracilaria tikvahiae* was able to outcompete *Ulva lactuca* in mixed macroalgal beds, despite the latter possessing a more rapid N uptake rate. Furthermore, *Gracilaria vermiculophylla*, normally found in the West Pacific, has invaded northern European estuaries as early as 2002, and has become among the most abundant macroalgae in the region, despite competition with *Ulva* and other ephemeral algae^56^. Despite these instances, were nutrients continuously added during experiments of the current study, it is plausible that the growth of *Ulva* would have been less affected by other autotrophs. Hence, the outcome of competition among estuarine autotrophs exposed to high CO_2_ depend, at least in part, on ambient N levels.

Dinoflagellates experienced more rapid growth when exposed to high CO_2_ while diatoms did not. Results from prior studies suggest that the response of plankton communities to elevated CO_2_ concentrations are likely to depend on the species present but that dinoflagellates are more prone to C-limitation than diatoms as dinoflagellates possess form II RubisCO, which has a low affinity for CO_2_^57, 58^. The dinoflagellates *Protoceratium reticulatum* ^59^, *Karlodinium veneficum* ^21^ and *Karenia brevis* ^60^ all grow more rapidly under high CO_2_ as do *Alexandrium* species from Europe (*Alexandrium minutum* ^61^; *Alexandrium ostenfeldii* ^22^ and the North America (*Alexandrium catenella* ^21, 62^; *Alexandrium fundyense* ^10^). While the general response of diatoms to elevated CO_2_ also appears to be species-specific, they seem to be generally less sensitive to changes in *p*CO_2_. Dozens of diatom species realize maximal growth rates under a wide range of pH/ *p*CO_2_ levels^27, 63–65^, although elevated CO_2_ enhances the growth rates of some species including *Pseudo-nitzschia fraudulenta* ^25^, *Pseudo-nitzschia multiseries* ^24^, and *Chaetoceros debilis* ^66^. Hence, the finding that CO_2_-stimulated growth of dinoflagellates but not diatoms are generally consistent with prior studies, but specific responses will depend on, among other factors, nutrient levels, the species of plankton present within a community, as well as competition with other autotrophs. Given dinoflagellates are responsible for most harmful algal blooms (HABs^67^) and that HABs are common within eutrophic settings^68^, the findings here suggest that high CO_2_, eutrophic estuaries may be more likely to host HABs with negative ecosystem consequences^10^.

Diatom and dinoflagellate growth rates were also affected by macroalgae with dinoflagellates growth being inhibited by *Gracilaria* but *Ulva* promoting the growth of diatoms. Prior studies have found that dinoflagellates in temperate estuaries are vulnerable to allelopathic inhibition by macroalgae^69, 70^ and *Gracilaria* spp. have been shown to allelopathically depress dinoflagellate growth rates^71, 72^. While *Ulva* has been found to allelopathically inhibit the growth of individual dinoflagellate species in culture^69^, during this study *Ulva* was found to have no effect on dinoflagellates but promoted the growth of diatoms. This finding indicates that *Ulva* may generally promote a succession within phytoplankton communities from dinoflagellates to diatoms, potentially via the remineralization of nutrients^73^ that promotes the growth of diatoms. The growth promotion of diatoms may be associated with the ability of *Ulva* to release and regenerate nutrients such as ammonium and phosphate^73, 74^. Another possibility is that vitamin B_12_-producing epiphytic bacteria on *Ulva* may have promoted the growth of diatoms. Diatoms are unable to synthesize vitamin B_12_ and as such, require bacteria for the production of the vitamin^75, 76^. Udell *et al*.^77^ found samples of *Ulva lactuca* in the same contiguous water body as the study site to be rich in vitamin B_12_ likely due to epiphytic bacteria. It is possible that the synthesis of vitamin B_12_ by epiphytic bacteria could have promoted the growth of diatoms in treatments containing *Ulva*.

There are numerous ecosystem implications of the overgrowth of macroalgae, such as *Ulva* and *Gracilaria*, due to the ability to outcompete autotrophs due to increased nutrient loading and CO_2_ concentrations. The overgrowth of bloom-forming macroalgae has been shown to have negative effects on seagrass meadows^29, 78^, kelp forests^79^, coral reefs^80, 81^ and even phytoplankton communities^69, 70, 72^. Although seagrass can experience enhanced growth in the presence of elevated CO_2_ concentrations^8^, increased nutrient loading favors macroalgal growth that can lead to the demise of seagrass^29, 82^ to the detriment of invertebrate and fish species that use seagrass for food, cover, and as nurseries^82–86^. The overgrowth of macroalgae can also directly cause mortality in some invertebrates^87–89^. Aside from the direct deleterious effects of ocean acidification on coral reefs and calcifying invertebrates^90^, continued eutrophication and ocean acidification may allow fast-growing macroalgae to overgrow substrate used by coral^91^. Adding to this point, Diaz-Pulido *et al*.^92^ found that the highest abundance of macroalgae on inshore reefs were species with CCM, but capable of using HCO_3_^−^ and CO_2_ for photosynthesis. If these algae are capable of increased growth under elevated CO_2_, it could potentially pose another threat to the future health of inshore reefs. However, offshore reefs may be more vulnerable to macroalgal overgrowth due to high abundances of macroalgae that strictly use CO_2_, which will directly benefit from elevated CO_2_ in the near future^92^. In sum, the benefits experienced by macroalgae as the result of increased CO_2_ concentrations can directly and indirectly harm a multitude of coastal ecosystems, as well as the organisms that reside within them.

Traditionally, nutrient loading has been considered a key factor structuring the dominance of autotrophs in estuaries, with seagrasses dominating estuaries with lower nutrient loads and phytoplankton outgrowing seagrasses and macroalgae in eutrophic systems with extended residence times^29^ (Fig. 8a). This and prior studies now allow the co-effect of CO_2_ to be considered in structuring autotrophic communities in estuaries. Among phytoplankton, dinoflagellates benefited more than diatoms from elevated CO_2_ during this and prior studies^10, 62^ (Fig. 8b) and *Ulva* and *Gracilaria* grow faster when exposed to elevated levels of CO_2_^11^ (Fig. 8b). When competition is considered, the ability of *Ulva* and *Gracilaria* to benefit from high CO_2_ and to inhibit the growth of competing phytoplankton via allelopathy, may allow macroalgae to dominate high nutrient, high CO_2_ estuaries^69, 70, 72^ (Fig. 8c). Under the single large dose of nutrients used in the experiments presented here, *Gracilaria* was the ultimate ‘winner’ within experimental treatments with high nutrients and high CO_2_ (Fig. 8c). Factors such as continuous nutrient loading and shading would likely alter the outcomes of competition.

## Electronic supplementary material

Supplementary Figure S1 and Tables S1-S4
