# Supplementary material for: The organizing effects of elevated CO2 on competition among estuarine primary producers
Source: Sci Rep. 2017 Aug 9;7:7667. doi: 10.1038/s41598-017-08178-5 (PMC5550435; doi:10.1038/s41598-017-08178-5)
Supplement: Supplementary file 1 — Supplementary Figure S1 and Tables S1-S4 [file 41598_2017_8178_MOESM1_ESM.pdf]

# The organizing effects of elevated $CO_2$ on competition among estuarine primary producers

**Craig S. Young[1] and Christopher J. Gobler[1,*]**

[1]Stony Brook University, School of Marine and Atmospheric Sciences, Southampton, NY 11968, USA

[*]Christopher.gobler@stonybrook.edu

**Supplementary Figure S1**

The $\delta^{13}C$ content of *Gracilaria* and *Ulva* exposed to elevated $CO_2$ concentrations compared to $\delta^{13}C$ signatures expected from the exclusive use of $HCO_3^-$ or $CO_2$. Box plots depict the mean median (line within the boxes), 25th and 75th percentiles (lower and upper edges of the boxes), and 10th and 90th percentiles of the data (lower and upper error bars).

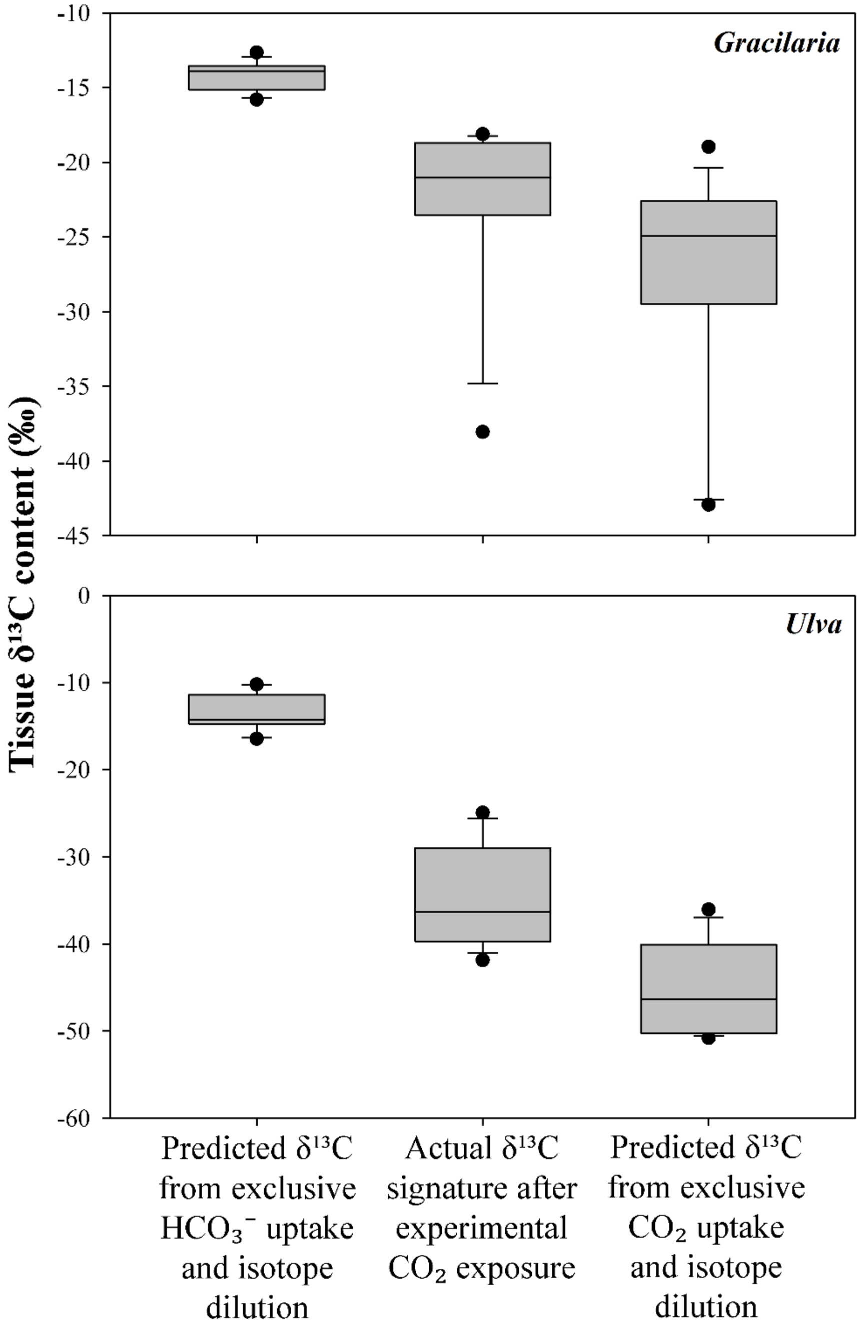

**Supplementary Tables S1**

Values of pH (NBS scale), salinity (g $kg^{-1}$), temperature (°C), and $pCO_2$ (µatm) for *Gracilaria* and *Ulva* for July through October experiments. Values represent means ± standard deviation.

***Ulva***

Early July

| Treatment | pH | Salinity | Temperature | $pCO_2$ |
|---|---|---|---|---|
| Ambient/Filtered | 8.23±0.11 | 31.0±1.4 | 16.9±0.9 | 210±20 |
| Ambient/Unfiltered | 8.38±0.10 | 30.0±0.0 | 17.1±0.9 | 190±10 |
| $CO_2$/Filtered | 7.19±0.10 | 31.0±1.4 | 16.3±0.7 | 2790±30 |
| $CO_2$/Unfiltered | 7.32±0.03 | 30.5±0.7 | 16.7±0.9 | 2580±60 |

Late July

| Treatment | pH | Salinity | Temperature | $pCO_2$ |
|---|---|---|---|---|
| Ambient/Filtered | 8.26±0.11 | 32.0±0.1 | 15.7±0.6 | 220±60 |
| Ambient/Unfiltered | 8.23±0.02 | 32.0±0.0 | 16.4±0.6 | 290±30 |
| $CO_2$/Filtered | 7.14±0.02 | 30.0±0.1 | 15.8±0.4 | 2760±20 |
| $CO_2$/Unfiltered | 7.21±0.02 | 32.0±0.0 | 16.5±0.2 | 3140±350 |

August

| Treatment | pH | Salinity | Temperature | $pCO_2$ |
|---|---|---|---|---|
| Ambient/Filtered | 8.10±0.04 | 29.4±0.1 | 17.9±2.6 | 290±50 |
| Ambient/Unfiltered | 8.22±0.02 | 29.4±0.3 | 17.5±3.3 | 260±20 |
| $CO_2$/Filtered | 7.10±0.01 | 29.3±0.1 | 17.1±1.1 | 2490±260 |
| $CO_2$/Unfiltered | 7.21±0.01 | 29.4±0.1 | 17.5±1.3 | 2990±100 |

September

| Treatment | pH | Salinity | Temperature | $pCO_2$ |
|---|---|---|---|---|
| Ambient/Filtered | 8.11±0.10 | 32.0±0.1 | 17.4±3.5 | 280±40 |
| Ambient/Unfiltered | 8.20±0.11 | 32.0±0.1 | 17.3±2.4 | 240±60 |
| $CO_2$/Filtered | 7.11±0.10 | 31.0±0.1 | 15.0±0.4 | 3090±230 |
| $CO_2$/Unfiltered | 7.20±0.02 | 32.0±0.2 | 14.8±0.1 | 2840±10 |

October

| Treatment | pH | Salinity | Temperature | $pCO_2$ |
|---|---|---|---|---|
| Ambient/Filtered | 8.02±0.02 | 30.0±0.1 | 15.0±0.7 | 350±30 |
| Ambient/Unfiltered | 8.10±0.02 | 29.9±0.0 | 14.0±0.7 | 350±1 |
| $CO_2$/Filtered | 7.32±0.01 | 30.1±0.2 | 14.2±1.7 | 1890±210 |
| $CO_2$/Unfiltered | 7.38±0.03 | 30.0±0.0 | 14.0±1.0 | 1760±180 |

***Gracilaria***

Early July

| Treatment | pH | Salinity | Temperature | $pCO_2$ |
|---|---|---|---|---|
| Ambient/Filtered | 8.19±0.15 | 31.5±2.1 | 16.1±0.6 | 230±10 |
| Ambient/Unfiltered | 8.35±0.10 | 30.0±0.1 | 17.8±1.3 | 210±10 |
| $CO_2$/Filtered | 7.18±0.13 | 31.0±1.4 | 15.2±0.6 | 2850±160 |
| $CO_2$/Unfiltered | 7.32±0.12 | 30.0±0.1 | 15.4±0.6 | 2370±170 |

Late July

| Treatment | pH | Salinity | Temperature | $pCO_2$ |
|---|---|---|---|---|
| Ambient/Filtered | 8.17±0.01 | 32.0±0.1 | 15.7±0.6 | 270±20 |
| Ambient/Unfiltered | 8.19±0.04 | 32.0±0.1 | 15.8±0.1 | 300±40 |
| $CO_2$/Filtered | 7.17±0.01 | 30.0±0.2 | 14.8±0.1 | 3380±220 |
| $CO_2$/Unfiltered | 7.20±0.02 | 32.0±0.1 | 15.1±0.1 | 3150±10 |

August

| Treatment | pH | Salinity | Temperature | $pCO_2$ |
|---|---|---|---|---|
| Ambient/Filtered | 8.12±0.07 | 29.4±0.1 | 17.8±2.7 | 280±60 |
| Ambient/Unfiltered | 8.19±0.02 | 29.4±0.5 | 17.8±2.8 | 260±10 |
| $CO_2$/Filtered | 7.08±0.02 | 29.3±0.2 | 17.0±1.3 | 2470±240 |
| $CO_2$/Unfiltered | 7.20±0.01 | 29.3±0.1 | 17.3±1.6 | 3000±20 |

September

| Treatment | pH | Salinity | Temperature | $pCO_2$ |
|---|---|---|---|---|
| Ambient/Filtered | 8.04±0.04 | 31.5±0.2 | 16.6±3.8 | 370±20 |
| Ambient/Unfiltered | 8.16±0.02 | 32.0±0.2 | 16.7±4.1 | 410±100 |
| $CO_2$/Filtered | 7.11±0.11 | 31.5±0.1 | 13.3±0.4 | 2850±10 |
| $CO_2$/Unfiltered | 7.18±0.03 | 32.0±0.0 | 15.3±0.2 | 2500±490 |

October

| Treatment | pH | Salinity | Temperature | $pCO_2$ |
|---|---|---|---|---|
| Ambient/Filtered | 8.00±0.01 | 30.2±0.0 | 13.6±0.8 | 370±20 |
| Ambient/Unfiltered | 8.07±0.02 | 30.0±0.1 | 14.5±0.8 | 390±10 |
| $CO_2$/Filtered | 7.31±0.02 | 30.1±0.1 | 14.4±0.4 | 1800±220 |
| $CO_2$/Unfiltered | 7.39±0.01 | 30.0±0.0 | 14.2±2.5 | 1750±50 |

***Gracilaria* and *Ulva***

Early July

| Treatment | pH | Salinity | Temperature | $pCO_2$ |
|---|---|---|---|---|
| Ambient/Filtered | 8.25±0.10 | 31.0±1.4 | 16.6±0.6 | 210±10 |
| Ambient/Unfiltered | 8.45±0.11 | 30.0±0.1 | 17.0±0.6 | 160±10 |
| $CO_2$/Filtered | 7.18±0.12 | 31.5±2.1 | 16.3±0.8 | 2800±60 |
| $CO_2$/Unfiltered | 7.33±0.10 | 30.0±0.0 | 16.4±0.6 | 2740±110 |

Late July

| Treatment | pH | Salinity | Temperature | $pCO_2$ |
|---|---|---|---|---|
| Ambient/Filtered | 8.23±0.09 | 32.0±0.2 | 15.7±1.1 | 230±60 |
| Ambient/Unfiltered | 8.22±0.04 | 32.0±0.1 | 15.6±0.6 | 290±40 |
| $CO_2$/Filtered | 7.13±0.02 | 30.0±0.0 | 15.2±1.3 | 2610±10 |
| $CO_2$/Unfiltered | 7.22±0.01 | 32.0±0.1 | 15.6±0.8 | 2950±80 |

August

| Treatment | pH | Salinity | Temperature | $pCO_2$ |
|---|---|---|---|---|
| Ambient/Filtered | 8.07±0.01 | 29.4±0.1 | 17.9±2.6 | 310±20 |
| Ambient/Unfiltered | 8.22±0.02 | 29.4±0.1 | 17.7±3.0 | 270±10 |
| $CO_2$/Filtered | 7.09±0.01 | 29.3±0.1 | 17.0±1.3 | 2435±190 |
| $CO_2$/Unfiltered | 7.20±0.01 | 29.4±0.1 | 17.3±1.6 | 3000±70 |

September

| Treatment | pH | Salinity | Temperature | $pCO_2$ |
|---|---|---|---|---|
| Ambient/Filtered | 8.14±0.01 | 32.0±0.1 | 17.8±3.0 | 250±40 |
| Ambient/Unfiltered | 8.13±0.02 | 31.5±0.0 | 16.4±3.8 | 320±50 |
| $CO_2$/Filtered | 7.11±0.10 | 31.5±0.5 | 15.0±0.8 | 2890±160 |
| $CO_2$/Unfiltered | 7.19±0.03 | 31.5±0.1 | 15.5±0.1 | 3000±190 |

October

| Treatment | pH | Salinity | Temperature | p$CO_2$ |
|---|---|---|---|---|
| Ambient/Filtered | 8.05±0.05 | 30.0±0.1 | 14.2±0.7 | 330±20 |
| Ambient/Unfiltered | 8.09±0.01 | 29.9±0.2 | 15.0±0.9 | 370±10 |
| $CO_2$/Filtered | 7.31±0.02 | 30.1±0.1 | 14.4±1.6 | 1850±290 |
| $CO_2$/Unfiltered | 7.39±0.05 | 29.9±0.2 | 14.3±2.2 | 1820±110 |

**Supplementary Tables S2**

Statistical analyses of variance for laboratory and *in situ* experiments (July through October 2015) for *Gracilaria* and *Ulva*.

Three-way analysis of variance for *Gracilaria* growth for July through October experiments

| Source of Variation | DF | SS | MS | F | P |
|---|---|---|---|---|---|
| $CO_2$ | 1 | 0.00731 | 0.00731 | 7.001 | 0.009 |
| Filtered | 1 | 0.000136 | 0.000136 | 0.13 | 0.719 |
| Competition | 1 | 0.000249 | 0.000249 | 0.239 | 0.626 |
| $CO_2$ x Filtered | 1 | 0.000117 | 0.000117 | 0.112 | 0.739 |
| $CO_2$ x Competition | 1 | 0.000126 | 0.000126 | 0.12 | 0.729 |
| Filtered x Competition | 1 | 0.000368 | 0.000368 | 0.352 | 0.554 |
| $CO_2$ x Filtered x Competition | 1 | 0.000181 | 0.000181 | 0.173 | 0.678 |
| Residual | 128 | 0.134 | 0.00104 | | |
| Total | 135 | 0.142 | 0.00105 | | |

Three-way analysis of variance for *Ulva* growth for July through October experiments

| Source of Variation | DF | SS | MS | F | P |
|---|---|---|---|---|---|
| $CO_2$ | 1 | 0.0832 | 0.0832 | 17.696 | <0.001 |
| Filtered | 1 | 0.00881 | 0.00881 | 1.876 | 0.173 |
| Competition | 1 | 0.0413 | 0.0413 | 8.793 | 0.004 |
| $CO_2$ x Filtered | 1 | 0.00218 | 0.00218 | 0.464 | 0.497 |
| $CO_2$ x Competition | 1 | 0.00619 | 0.00619 | 1.316 | 0.253 |
| Filtered x Competition | 1 | 0.0112 | 0.0112 | 2.393 | 0.124 |
| $CO_2$ x Filtered x Competition | 1 | 0.000885 | 0.000885 | 0.188 | 0.665 |
| Residual | 124 | 0.583 | 0.0047 | | |
| Total | 131 | 0.739 | 0.00564 | | |

One-way analysis of variance for *Gracilaria* growth under control and *in situ* conditions

| Source of Variation | DF | SS | MS | F | P |
|---|---|---|---|---|---|
| Between Groups | 4 | 0.000459 | 0.000115 | 0.0526 | 0.995 |
| Residual | 79 | 0.172 | 0.00218 | | |
| Total | 83 | 0.173 | | | |

One-way analysis of variance for *Ulva* growth under control and *in situ* conditions

| Source of Variation | DF | SS | MS | F | P |
|---|---|---|---|---|---|
| Between Groups | 4 | 0.0362 | 0.00905 | 1.979 | 0.106 |
| Residual | 78 | 0.357 | 0.00457 | | |
| Total | 82 | 0.393 | | | |

Three-way analysis of variance for *Ulva* growth for the early July experiment

| Source of Variation | DF | SS | MS | F | P |
|---|---|---|---|---|---|
| $CO_2$ | 1 | 0.0106 | 0.0106 | 9.157 | 0.009 |
| Filtered | 1 | 0.0105 | 0.0105 | 9.069 | 0.009 |
| Competition | 1 | 0.0109 | 0.0109 | 9.43 | 0.008 |
| $CO_2$ x Filtered | 1 | 0.0000119 | 0.0000119 | 0.0103 | 0.92 |
| $CO_2$ x Competition | 1 | 0.00304 | 0.00304 | 2.622 | 0.126 |
| Filtered x Competition | 1 | 0.00868 | 0.00868 | 7.494 | 0.015 |
| $CO_2$ x Filtered x Competition | 1 | 0.00134 | 0.00134 | 1.16 | 0.298 |
| Residual | 15 | 0.0174 | 0.00116 | | |
| Total | 22 | 0.065 | 0.00296 | | |

Three-way analysis of variance for *Ulva* growth for the late July experiment

| Source of Variation | DF | SS | MS | F | P |
|---|---|---|---|---|---|
| $CO_2$ | 1 | 0.0933 | 0.0933 | 11.504 | 0.004 |
| Filtered | 1 | 0.122 | 0.122 | 15.017 | 0.001 |
| Competition | 1 | 0.00184 | 0.00184 | 0.227 | 0.64 |
| $CO_2$ x Filtered | 1 | 0.0172 | 0.0172 | 2.12 | 0.165 |
| $CO_2$ x Competition | 1 | 0.00137 | 0.00137 | 0.169 | 0.687 |
| Filtered x Competition | 1 | 0.00985 | 0.00985 | 1.215 | 0.287 |
| $CO_2$ x Filtered x Competition | 1 | 0.00781 | 0.00781 | 0.963 | 0.341 |
| Residual | 16 | 0.13 | 0.00811 | | |
| Total | 23 | 0.383 | 0.0166 | | |

Three-way analysis of variance for *Ulva* growth for the August experiment

| Source of Variation | DF | SS | MS | F | P |
|---|---|---|---|---|---|
| $CO_2$ | 1 | 0.0115 | 0.0115 | 5.908 | 0.027 |
| Filtered | 1 | 0.00238 | 0.00238 | 1.226 | 0.285 |
| Competition | 1 | 0.0089 | 0.0089 | 4.576 | 0.048 |
| $CO_2$ x Filtered | 1 | 0.000283 | 0.000283 | 0.145 | 0.708 |
| $CO_2$ x Competition | 1 | 0.0000132 | 0.0000132 | 0.00679 | 0.935 |
| Filtered x Competition | 1 | 1.21E-06 | 1.21E-06 | 0.000625 | 0.98 |
| $CO_2$ x Filtered x Competition | 1 | 0.00357 | 0.00357 | 1.834 | 0.194 |
| Residual | 16 | 0.0311 | 0.00195 | | |
| Total | 23 | 0.0578 | 0.00251 | | |

Three-way analysis of variance for *Ulva* growth for the September experiment

| Source of Variation | DF | SS | MS | F | P |
|---|---|---|---|---|---|
| $CO_2$ | 1 | 0.0582 | 0.0582 | 12.559 | 0.002 |
| Filtered | 1 | 0.00245 | 0.00245 | 0.528 | 0.475 |
| Competition | 1 | 0.0114 | 0.0114 | 2.456 | 0.131 |
| $CO_2$ x Filtered | 1 | 0.0256 | 0.0256 | 5.518 | 0.028 |
| $CO_2$ x Competition | 1 | 0.00139 | 0.00139 | 0.3 | 0.589 |
| Filtered x Competition | 1 | 0.00411 | 0.00411 | 0.886 | 0.356 |
| $CO_2$ x Filtered x Competition | 1 | 0.00834 | 0.00834 | 1.8 | 0.193 |
| Residual | 23 | 0.107 | 0.00464 | | |
| Total | 30 | 0.223 | 0.00742 | | |

Three-way analysis of variance for *Ulva* growth for the October experiment

| Source of Variation | DF | SS | MS | F | P |
|---|---|---|---|---|---|
| $CO_2$ | 1 | 0.00109 | 0.00109 | 0.461 | 0.503 |
| Filtered | 1 | 0.00423 | 0.00423 | 1.786 | 0.194 |
| Competition | 1 | 0.00348 | 0.00348 | 1.469 | 0.237 |
| $CO_2$ x Filtered | 1 | 0.000394 | 0.000394 | 0.167 | 0.687 |
| $CO_2$ x Competition | 1 | 0.0000488 | 0.0000488 | 0.0206 | 0.887 |
| Filtered x Competition | 1 | 0.000529 | 0.000529 | 0.224 | 0.641 |
| $CO_2$ x Filtered x Competition | 1 | 0.000128 | 0.000128 | 0.0539 | 0.818 |
| Residual | 24 | 0.0568 | 0.00237 | | |
| Total | 31 | 0.0667 | 0.00215 | | |

Three-way analysis of variance for *Gracilaria* growth for the early July experiment

| Source of Variation | DF | SS | MS | F | P |
|---|---|---|---|---|---|
| $CO_2$ | 1 | 0.000941 | 0.000941 | 1.304 | 0.27 |
| Filtered | 1 | 0.00555 | 0.00555 | 7.697 | 0.014 |
| Competition | 1 | 0.000154 | 0.000154 | 0.213 | 0.651 |
| $CO_2$ x Filtered | 1 | 0.00149 | 0.00149 | 2.065 | 0.17 |
| $CO_2$ x Competition | 1 | 0.0000196 | 0.0000196 | 0.0272 | 0.871 |
| Filtered x Competition | 1 | 0.0000531 | 0.0000531 | 0.0736 | 0.79 |
| $CO_2$ x Filtered x Competition | 1 | 0.00128 | 0.00128 | 1.77 | 0.202 |
| Residual | 16 | 0.0115 | 0.000722 | | |
| Total | 23 | 0.021 | 0.000915 | | |

Three-way analysis of variance for *Gracilaria* growth for the late July experiment

| Source of Variation | DF | SS | MS | F | P |
|---|---|---|---|---|---|
| $CO_2$ | 1 | 0.00206 | 0.00206 | 5.088 | 0.039 |
| Filtered | 1 | 9.42E-06 | 9.42E-06 | 0.0233 | 0.881 |
| Competition | 1 | 0.000556 | 0.000556 | 1.373 | 0.26 |
| $CO_2$ x Filtered | 1 | 0.000666 | 0.000666 | 1.643 | 0.219 |
| $CO_2$ x Competition | 1 | 0.000525 | 0.000525 | 1.295 | 0.273 |
| Filtered x Competition | 1 | 0.0000027 | 0.0000027 | 0.00667 | 0.936 |
| $CO_2$ x Filtered x Competition | 1 | 0.0000374 | 0.0000374 | 0.0924 | 0.765 |
| Residual | 15 | 0.00608 | 0.000405 | | |
| Total | 22 | 0.00989 | 0.000449 | | |

Three-way analysis of variance for *Gracilaria* growth for the August experiment

| Source of Variation | DF | SS | MS | F | P |
|---|---|---|---|---|---|
| $CO_2$ | 1 | 0.000999 | 0.000999 | 5.763 | 0.031 |
| Filtered | 1 | 0.0000351 | 0.0000351 | 0.203 | 0.659 |
| Competition | 1 | 0.000141 | 0.000141 | 0.811 | 0.383 |
| $CO_2$ x Filtered | 1 | 0.000114 | 0.000114 | 0.658 | 0.431 |
| $CO_2$ x Competition | 1 | 0.000234 | 0.000234 | 1.351 | 0.265 |
| Filtered x Competition | 1 | 0.00131 | 0.00131 | 7.563 | 0.016 |
| $CO_2$ x Filtered x Competition | 1 | 0.0000623 | 0.0000623 | 0.359 | 0.558 |
| Residual | 14 | 0.00243 | 0.000173 | | |
| Total | 21 | 0.00524 | 0.00025 | | |

Three-way analysis of variance for *Gracilaria* growth for the September experiment

| Source of Variation | DF | SS | MS | F | P |
|---|---|---|---|---|---|
| $CO_2$ | 1 | 0.00132 | 0.00132 | 2.466 | 0.129 |
| Filtered | 1 | 0.000429 | 0.000429 | 0.798 | 0.381 |
| Competition | 1 | 0.000297 | 0.000297 | 0.553 | 0.464 |
| $CO_2$ x Filtered | 1 | 0.0000624 | 0.0000624 | 0.116 | 0.736 |
| $CO_2$ x Competition | 1 | 8.93E-06 | 8.93E-06 | 0.0166 | 0.899 |
| Filtered x Competition | 1 | 0.000307 | 0.000307 | 0.571 | 0.457 |
| $CO_2$ x Filtered x Competition | 1 | 0.0000483 | 0.0000483 | 0.0898 | 0.767 |
| Residual | 24 | 0.0129 | 0.000537 | | |
| Total | 31 | 0.0154 | 0.000496 | | |

Three-way analysis of variance for *Gracilaria* growth for the October experiment

| Source of Variation | DF | SS | MS | F | P |
|---|---|---|---|---|---|
| $CO_2$ | 1 | 0.00257 | 0.00257 | 6.01 | 0.022 |
| Filtered | 1 | 0.000228 | 0.000228 | 0.532 | 0.473 |
| Competition | 1 | 0.00129 | 0.00129 | 3.007 | 0.096 |
| $CO_2$ x Filtered | 1 | 0.000257 | 0.000257 | 0.599 | 0.447 |
| $CO_2$ x Competition | 1 | 4.51E-07 | 4.51E-07 | 0.00105 | 0.974 |
| Filtered x Competition | 1 | 1.01E-07 | 1.01E-07 | 0.000236 | 0.988 |
| $CO_2$ x Filtered x Competition | 1 | 2.31E-06 | 2.31E-06 | 0.0054 | 0.942 |
| Residual | 24 | 0.0103 | 0.000428 | | |
| Total | 31 | 0.0146 | 0.000472 | | |

Three-way analysis of variance for diatom growth for the September experiment

| Source of Variation | DF | SS | MS | F | P |
|---|---|---|---|---|---|
| $CO_2$ | 1 | 0.00127 | 0.00127 | 0.139 | 0.712 |
| *Ulva* | 1 | 0.047 | 0.047 | 5.17 | 0.032 |
| *Gracilaria* | 1 | 0.00542 | 0.00542 | 0.596 | 0.448 |
| $CO_2$ x *Ulva* | 1 | 0.00513 | 0.00513 | 0.565 | 0.46 |
| $CO_2$ x *Gracilaria* | 1 | 0.0196 | 0.0196 | 2.159 | 0.155 |
| *Ulva* x *Gracilaria* | 1 | 0.000237 | 0.000237 | 0.0261 | 0.873 |
| $CO_2$ x *Ulva* x *Gracilaria* | 1 | 0.0075 | 0.0075 | 0.825 | 0.373 |
| Residual | 24 | 0.218 | 0.00909 | | |
| Total | 31 | 0.304 | 0.00982 | | |

Three-way analysis of variance for dinoflagellate growth for the September experiment

| Source of Variation | DF | SS | MS | F | P |
|---|---|---|---|---|---|
| $CO_2$ | 1 | 0.00347 | 0.00347 | 2.203 | 0.151 |
| *Ulva* | 1 | 0.00354 | 0.00354 | 2.246 | 0.147 |
| *Gracilaria* | 1 | 0.0403 | 0.0403 | 25.611 | <0.001 |
| $CO_2$ x *Ulva* | 1 | 0.00154 | 0.00154 | 0.975 | 0.333 |
| $CO_2$ x *Gracilaria* | 1 | 0.00316 | 0.00316 | 2.005 | 0.17 |
| *Ulva* x *Gracilaria* | 1 | 0.00291 | 0.00291 | 1.849 | 0.187 |
| $CO_2$ x *Ulva* x *Gracilaria* | 1 | 0.0000306 | 0.0000306 | 0.0194 | 0.89 |
| Residual | 24 | 0.0378 | 0.00158 | | |
| Total | 31 | 0.0928 | 0.00299 | | |

One-way analysis of variance for changes in diatom abundance in elevated $CO_2$ treatments during the September experiment

| Source of Variation | DF | SS | MS | F | P |
|---|---|---|---|---|---|
| Between Groups | 1 | 20930450 | 20930450 | 44.91 | <0.001 |
| Residual | 6 | 2796350 | 466058.33 | | |
| Total | 7 | 23726800 | | | |

One-way analysis of variance for changes in dinoflagellate abundance in elevated $CO_2$ treatments during the September experiment

| Source of Variation | DF | SS | MS | F | P |
|---|---|---|---|---|---|
| Between Groups | 1 | 2420000 | 2420000 | 10.204 | 0.019 |
| Residual | 6 | 1423000 | 237166.67 | | |
| Total | 7 | 3843000 | | | |

One-way analysis of variance for changes in diatom abundance in ambient treatments during the September experiment

| Source of Variation | DF | SS | MS | F | P |
|---|---|---|---|---|---|
| Between Groups | 1 | 7761800 | 7761800 | 12.669 | 0.012 |
| Residual | 6 | 3675950 | 612658.33 | | |
| Total | 7 | 11437750 | | | |

One-way analysis of variance for changes in dinoflagellate abundance in ambient treatments during the September experiment

| Source of Variation | DF | SS | MS | F | P |
|---|---|---|---|---|---|
| Between Groups | 1 | 11685344 | 11685344 | 17.003 | 0.009 |
| Residual | 5 | 3436341.7 | 687268.33 | | |
| Total | 6 | 15121686 | | | |

One-way analysis of variance for changes in diatom abundance in elevated $CO_2$ treatments during the October experiment

| Source of Variation | DF | SS | MS | F | P |
|---|---|---|---|---|---|
| Between Groups | 1 | 30186450 | 30186450 | 20.589 | 0.004 |
| Residual | 6 | 8796700 | 1466116.7 | | |
| Total | 7 | 38983150 | | | |

One-way analysis of variance for changes in dinoflagellate abundance in elevated $CO_2$ treatments during the October experiment

| Source of Variation | DF | SS | MS | F | P |
|---|---|---|---|---|---|
| Between Groups | 1 | 13912813 | 13912813 | 73.452 | <0.001 |
| Residual | 6 | 1136475 | 189412.5 | | |
| Total | 7 | 15049288 | | | |

One-way analysis of variance for changes in diatom abundance in ambient treatments during the October experiment

| Source of Variation | DF | SS | MS | F | P |
|---|---|---|---|---|---|
| Between Groups | 1 | 35490313 | 35490313 | 61.082 | <0.001 |
| Residual | 6 | 3486175 | 581029.17 | | |
| Total | 7 | 38976488 | | | |

One-way analysis of variance for changes in dinoflagellate abundance in ambient treatments during the October experiment

| Source of Variation | DF | SS | MS | F | P |
|---|---|---|---|---|---|
| Between Groups | 1 | 48856613 | 48856613 | 38.12 | <0.001 |
| Residual | 6 | 7689875 | 1281645.8 | | |
| Total | 7 | 56546488 | | | |

Three-way analysis of variance of the tissue $\delta^{13}C$ for *Ulva* for the early July experiment

| Source of Variation | DF | SS | MS | F | P |
|---|---|---|---|---|---|
| $CO_2$ | 1 | 3287.232 | 3287.232 | 217.325 | <0.001 |
| *Gracilaria* | 1 | 70.658 | 70.658 | 4.671 | 0.046 |
| Filtered | 1 | 0.06 | 0.06 | 0.00397 | 0.951 |
| $CO_2$ x *Gracilaria* | 1 | 2.968 | 2.968 | 0.196 | 0.664 |
| $CO_2$ x Filtered | 1 | 0.443 | 0.443 | 0.0293 | 0.866 |
| *Gracilaria* x Filtered | 1 | 121.5 | 121.5 | 8.033 | 0.012 |
| $CO_2$ x *Gracilaria* x Filtered | 1 | 87.937 | 87.937 | 5.814 | 0.028 |
| Residual | 16 | 242.014 | 15.126 | | |
| Total | 23 | 3812.812 | 165.774 | | |

Three-way analysis of variance of the tissue $\delta^{13}C$ for *Ulva* for the late July experiment

| Source of Variation | DF | SS | MS | F | P |
|---|---|---|---|---|---|
| $CO_2$ | 1 | 4319.63 | 4319.63 | 359.192 | <0.001 |
| *Gracilaria* | 1 | 1.197 | 1.197 | 0.0995 | 0.756 |
| Filtered | 1 | 11.399 | 11.399 | 0.948 | 0.345 |
| $CO_2$ x *Gracilaria* | 1 | 1.197 | 1.197 | 0.0995 | 0.756 |
| $CO_2$ x Filtered | 1 | 52.747 | 52.747 | 4.386 | 0.053 |
| *Gracilaria* x Filtered | 1 | 1.52 | 1.52 | 0.126 | 0.727 |
| $CO_2$ x *Gracilaria* x Filtered | 1 | 13.321 | 13.321 | 1.108 | 0.308 |
| Residual | 16 | 192.415 | 12.026 | | |
| Total | 23 | 4593.426 | 199.714 | | |

Three-way analysis of variance of the tissue $\delta^{13}C$ for *Ulva* for the August experiment

| Source of Variation | DF | SS | MS | F | P |
|---|---|---|---|---|---|
| $CO_2$ | 1 | 2026.395 | 2026.395 | 273.867 | <0.001 |
| *Gracilaria* | 1 | 2.142 | 2.142 | 0.289 | 0.598 |
| Filtered | 1 | 75.935 | 75.935 | 10.263 | 0.006 |
| $CO_2$ x *Gracilaria* | 1 | 1.832 | 1.832 | 0.248 | 0.626 |
| $CO_2$ x Filtered | 1 | 1.744 | 1.744 | 0.236 | 0.634 |
| *Gracilaria* x Filtered | 1 | 4.208 | 4.208 | 0.569 | 0.462 |
| $CO_2$ x *Gracilaria* x Filtered | 1 | 5.273 | 5.273 | 0.713 | 0.411 |
| Residual | 16 | 118.387 | 7.399 | | |
| Total | 23 | 2235.917 | 97.214 | | |

Three-way analysis of variance of the tissue $\delta^{13}C$ for *Ulva* for the September experiment

| Source of Variation | DF | SS | MS | F | P |
|---|---|---|---|---|---|
| $CO_2$ | 1 | 2195.378 | 2195.378 | 667.221 | <0.001 |
| *Gracilaria* | 1 | 9.573 | 9.573 | 2.909 | 0.102 |
| Filtered | 1 | 27.114 | 27.114 | 8.241 | 0.009 |
| $CO_2$ x *Gracilaria* | 1 | 0.667 | 0.667 | 0.203 | 0.657 |
| $CO_2$ x Filtered | 1 | 10.879 | 10.879 | 3.306 | 0.082 |
| *Gracilaria* x Filtered | 1 | 0.781 | 0.781 | 0.237 | 0.631 |
| $CO_2$ x *Gracilaria* x Filtered | 1 | 2.813 | 2.813 | 0.855 | 0.365 |
| Residual | 23 | 75.678 | 3.29 | | |
| Total | 30 | 2378.782 | 79.293 | | |

Three-way analysis of variance of the tissue $\delta^{13}C$ for *Ulva* for the October experiment

| Source of Variation | DF | SS | MS | F | P |
|---|---|---|---|---|---|
| $CO_2$ | 1 | 3886.313 | 3886.313 | 265.278 | <0.001 |
| *Gracilaria* | 1 | 5.977 | 5.977 | 0.408 | 0.529 |
| Filtered | 1 | 1.562 | 1.562 | 0.107 | 0.747 |
| $CO_2$ x *Gracilaria* | 1 | 11.198 | 11.198 | 0.764 | 0.391 |
| $CO_2$ x Filtered | 1 | 10.615 | 10.615 | 0.725 | 0.403 |
| *Gracilaria* x Filtered | 1 | 23.822 | 23.822 | 1.626 | 0.214 |
| $CO_2$ x *Gracilaria* x Filtered | 1 | 30.988 | 30.988 | 2.115 | 0.159 |
| Residual | 24 | 351.599 | 14.65 | | |
| Total | 31 | 4322.075 | 139.422 | | |

Three-way analysis of variance of the tissue $\delta^{13}C$ for *Gracilaria* for the early July experiment

| Source of Variation | DF | SS | MS | F | P |
|---|---|---|---|---|---|
| $CO_2$ | 1 | 2005.865 | 2005.865 | 456.536 | <0.001 |
| *Ulva* | 1 | 1.799 | 1.799 | 0.409 | 0.531 |
| Filtered | 1 | 13.039 | 13.039 | 2.968 | 0.104 |
| $CO_2$ x *Ulva* | 1 | 11.551 | 11.551 | 2.629 | 0.124 |
| $CO_2$ x Filtered | 1 | 0.746 | 0.746 | 0.17 | 0.686 |
| *Ulva* x Filtered | 1 | 25.523 | 25.523 | 5.809 | 0.028 |
| $CO_2$ x *Ulva* x Filtered | 1 | 20.963 | 20.963 | 4.771 | 0.044 |
| Residual | 16 | 70.299 | 4.394 | | |
| Total | 23 | 2149.783 | 93.469 | | |

Three-way analysis of variance of the tissue $\delta^{13}C$ for *Gracilaria* for the late July experiment

| Source of Variation | DF | SS | MS | F | P |
|---|---|---|---|---|---|
| $CO_2$ | 1 | 807.244 | 807.244 | 62.43 | <0.001 |
| *Ulva* | 1 | 16.187 | 16.187 | 1.252 | 0.28 |
| Filtered | 1 | 33.868 | 33.868 | 2.619 | 0.125 |
| $CO_2$ x *Ulva* | 1 | 14.837 | 14.837 | 1.147 | 0.3 |
| $CO_2$ x Filtered | 1 | 1.515 | 1.515 | 0.117 | 0.737 |
| *Ulva* x Filtered | 1 | 5.772 | 5.772 | 0.446 | 0.514 |
| $CO_2$ x *Ulva* x Filtered | 1 | 4.412 | 4.412 | 0.341 | 0.567 |
| Residual | 16 | 206.885 | 12.93 | | |
| Total | 23 | 1090.719 | 47.423 | | |

Three-way analysis of variance of the tissue $\delta^{13}C$ for *Gracilaria* for the August experiment

| Source of Variation | DF | SS | MS | F | P |
|---|---|---|---|---|---|
| $CO_2$ | 1 | 307.459 | 307.459 | 266.635 | <0.001 |
| *Ulva* | 1 | 2.262 | 2.262 | 1.962 | 0.182 |
| Filtered | 1 | 2.256 | 2.256 | 1.957 | 0.182 |
| $CO_2$ x *Ulva* | 1 | 0.00217 | 0.00217 | 0.00188 | 0.966 |
| $CO_2$ x Filtered | 1 | 7.398 | 7.398 | 6.416 | 0.023 |
| *Ulva* x Filtered | 1 | 0.487 | 0.487 | 0.423 | 0.525 |
| $CO_2$ x *Ulva* x Filtered | 1 | 9.983 | 9.983 | 8.657 | 0.01 |
| Residual | 15 | 17.297 | 1.153 | | |
| Total | 22 | 360.766 | 16.398 | | |

Three-way analysis of variance of the tissue $\delta^{13}C$ for *Gracilaria* for the September experiment

| Source of Variation | DF | SS | MS | F | P |
|---|---|---|---|---|---|
| $CO_2$ | 1 | 260.833 | 260.833 | 266.558 | <0.001 |
| *Ulva* | 1 | 0.0242 | 0.0242 | 0.0247 | 0.876 |
| Filtered | 1 | 0.324 | 0.324 | 0.331 | 0.57 |
| $CO_2$ x *Ulva* | 1 | 0.461 | 0.461 | 0.471 | 0.499 |
| $CO_2$ x Filtered | 1 | 0.019 | 0.019 | 0.0194 | 0.89 |
| *Ulva* x Filtered | 1 | 0.183 | 0.183 | 0.187 | 0.669 |
| $CO_2$ x *Ulva* x Filtered | 1 | 0.316 | 0.316 | 0.323 | 0.575 |
| Residual | 24 | 23.485 | 0.979 | | |
| Total | 31 | 285.644 | 9.214 | | |

Three-way analysis of variance of the tissue $\delta^{13}C$ for *Gracilaria* for the October experiment

| Source of Variation | DF | SS | MS | F | P |
|---|---|---|---|---|---|
| $CO_2$ | 1 | 885.354 | 885.354 | 399.976 | <0.001 |
| *Ulva* | 1 | 0.329 | 0.329 | 0.148 | 0.704 |
| Filtered | 1 | 2.032 | 2.032 | 0.918 | 0.348 |
| $CO_2$ x *Ulva* | 1 | 2.864 | 2.864 | 1.294 | 0.267 |
| $CO_2$ x Filtered | 1 | 0.833 | 0.833 | 0.376 | 0.546 |
| *Ulva* x Filtered | 1 | 9.388 | 9.388 | 4.241 | 0.051 |
| $CO_2$ x *Ulva* x Filtered | 1 | 6.562 | 6.562 | 2.965 | 0.099 |
| Residual | 23 | 50.911 | 2.214 | | |
| Total | 30 | 973.57 | 32.452 | | |

One-way analysis of variance of the $\delta^{13}C$ content of *Gracilaria* exposed to elevated $CO_2$ concentrations compared with the $\delta^{13}C$ content expected from the exclusive use of $CO_2$ or the exclusive use of $HCO_3^-$ (Fig. 4). Tukey tests were used to determine which groups were significantly different from one another.

| Source of Variation | DF | SS | MS | F | P |
|---|---|---|---|---|---|
| Between Groups | 2 | 1583.667 | 791.834 | 24.706 | <0.001 |
| Residual | 47 | 1506.357 | 32.05 | | |
| Total | 49 | 3090.024 | | | |

One-way analysis of variance of the $\delta^{13}C$ content of *Ulva* exposed to elevated $CO_2$ concentrations compared with the $\delta^{13}C$ content expected from the exclusive use of $CO_2$ or the exclusive use of $HCO_3^-$ (Fig. 4). Tukey tests were used to determine which groups were significantly different from one another.

| Source of Variation | DF | SS | MS | F | P |
|---|---|---|---|---|---|
| Between Groups | 2 | 8621.591 | 4310.796 | 195.973 | <0.001 |
| Residual | 48 | 1055.849 | 21.997 | | |
| Total | 50 | 9677.44 | | | |

Three-way analysis of variance of the tissue carbon for *Gracilaria* for the early July experiment

| Source of Variation | DF | SS | MS | F | P |
|---|---|---|---|---|---|
| $CO_2$ | 1 | 4.78E-05 | 4.78E-05 | 0.102 | 0.753 |
| *Ulva* | 1 | 9.93E-05 | 9.93E-05 | 0.213 | 0.651 |
| Filtered | 1 | 9.98E-05 | 9.98E-05 | 0.214 | 0.65 |
| $CO_2$ x *Ulva* | 1 | 8.59E-05 | 8.59E-05 | 0.184 | 0.674 |
| $CO_2$ x Filtered | 1 | 0.000302 | 0.000302 | 0.647 | 0.433 |
| *Ulva* x Filtered | 1 | 0.00264 | 0.00264 | 5.648 | 0.03 |
| $CO_2$ x *Ulva* x Filtered | 1 | 0.000032 | 0.000032 | 0.0685 | 0.797 |
| Residual | 16 | 0.00747 | 0.000467 | | |
| Total | 23 | 0.0108 | 0.000468 | | |

Three-way analysis of variance of the tissue carbon for *Gracilaria* for the late July experiment

| Source of Variation | DF | SS | MS | F | P |
|---|---|---|---|---|---|
| $CO_2$ | 1 | 0.00144 | 0.00144 | 5.247 | 0.036 |
| *Ulva* | 1 | 1.33E-05 | 1.33E-05 | 0.0486 | 0.828 |
| Filtered | 1 | 0.0026 | 0.0026 | 9.506 | 0.007 |
| $CO_2$ x *Ulva* | 1 | 0.00129 | 0.00129 | 4.722 | 0.045 |
| $CO_2$ x Filtered | 1 | 0.000171 | 0.000171 | 0.625 | 0.441 |
| *Ulva* x Filtered | 1 | 0.0019 | 0.0019 | 6.943 | 0.018 |
| $CO_2$ x *Ulva* x Filtered | 1 | 1.53E-05 | 1.53E-05 | 0.0559 | 0.816 |
| Residual | 16 | 0.00438 | 0.000274 | | |
| Total | 23 | 0.0118 | 0.000513 | | |

Three-way analysis of variance of the tissue carbon for *Gracilaria* for the August experiment

| Source of Variation | DF | SS | MS | F | P |
|---|---|---|---|---|---|
| $CO_2$ | 1 | 0.00139 | 0.00139 | 2.438 | 0.138 |
| *Ulva* | 1 | 0.000205 | 0.000205 | 0.36 | 0.557 |
| Filtered | 1 | 0.000139 | 0.000139 | 0.244 | 0.628 |
| $CO_2$ x *Ulva* | 1 | 9.57E-06 | 9.57E-06 | 0.0167 | 0.899 |
| $CO_2$ x Filtered | 1 | 0.000235 | 0.000235 | 0.41 | 0.531 |
| *Ulva* x Filtered | 1 | 0.00102 | 0.00102 | 1.788 | 0.2 |
| $CO_2$ x *Ulva* x Filtered | 1 | 3.93E-08 | 3.93E-08 | 6.89E-05 | 0.993 |
| Residual | 16 | 0.00914 | 0.000571 | | |
| Total | 23 | 0.0121 | 0.000528 | | |

Three-way analysis of variance of the tissue carbon for *Gracilaria* for the September experiment

| Source of Variation | DF | SS | MS | F | P |
|---|---|---|---|---|---|
| $CO_2$ | 1 | 0.00279 | 0.00279 | 3.026 | 0.095 |
| *Ulva* | 1 | 1.33E-05 | 1.33E-05 | 0.0144 | 0.905 |
| Filtered | 1 | 8.49E-06 | 8.49E-06 | 0.00921 | 0.924 |
| $CO_2$ x *Ulva* | 1 | 0.000177 | 0.000177 | 0.193 | 0.665 |
| $CO_2$ x Filtered | 1 | 9.27E-05 | 9.27E-05 | 0.101 | 0.754 |
| *Ulva* x Filtered | 1 | 1.39E-09 | 1.39E-09 | 1.51E-06 | 0.999 |
| $CO_2$ x *Ulva* x Filtered | 1 | 0.000142 | 0.000142 | 0.154 | 0.698 |
| Residual | 24 | 0.0221 | 0.000921 | | |
| Total | 31 | 0.0253 | 0.000817 | | |

Three-way analysis of variance of the tissue carbon for *Gracilaria* for the October experiment

| Source of Variation | DF | SS | MS | F | P |
|---|---|---|---|---|---|
| $CO_2$ | 1 | 0.000717 | 0.000717 | 1.115 | 0.302 |
| *Ulva* | 1 | 3.35E-05 | 3.35E-05 | 0.0521 | 0.821 |
| Filtered | 1 | 0.00121 | 0.00121 | 1.878 | 0.184 |
| $CO_2$ x *Ulva* | 1 | 3.29E-06 | 3.29E-06 | 0.00511 | 0.944 |
| $CO_2$ x Filtered | 1 | 0.000663 | 0.000663 | 1.031 | 0.32 |
| *Ulva* x Filtered | 1 | 5.55E-05 | 5.55E-05 | 0.0863 | 0.772 |
| $CO_2$ x *Ulva* x Filtered | 1 | 0.00232 | 0.00232 | 3.603 | 0.07 |
| Residual | 23 | 0.0148 | 0.000643 | | |
| Total | 30 | 0.0198 | 0.000661 | | |

Three-way analysis of variance of the tissue nitrogen for *Gracilaria* for the early July experiment

| Source of Variation | DF | SS | MS | F | P |
|---|---|---|---|---|---|
| $CO_2$ | 1 | 2.22E-05 | 2.22E-05 | 1.469 | 0.243 |
| *Ulva* | 1 | 6.4E-07 | 6.4E-07 | 0.0424 | 0.839 |
| Filtered | 1 | 0.000215 | 0.000215 | 14.231 | 0.002 |
| $CO_2$ x *Ulva* | 1 | 2.07E-05 | 2.07E-05 | 1.372 | 0.259 |
| $CO_2$ x Filtered | 1 | 4.88E-05 | 4.88E-05 | 3.236 | 0.091 |
| *Ulva* x Filtered | 1 | 0.000136 | 0.000136 | 9.014 | 0.008 |
| $CO_2$ x *Ulva* x Filtered | 1 | 2.57E-05 | 2.57E-05 | 1.705 | 0.21 |
| Residual | 16 | 0.000241 | 1.51E-05 | | |
| Total | 23 | 0.00071 | 3.09E-05 | | |

Three-way analysis of variance of the tissue nitrogen for *Gracilaria* for the late July experiment

| Source of Variation | DF | SS | MS | F | P |
|---|---|---|---|---|---|
| $CO_2$ | 1 | 1.82E-05 | 1.82E-05 | 3.86 | 0.067 |
| *Ulva* | 1 | 1.51E-05 | 1.51E-05 | 3.197 | 0.093 |
| Filtered | 1 | 0.000191 | 0.000191 | 40.666 | <0.001 |
| $CO_2$ x *Ulva* | 1 | 6.45E-05 | 6.45E-05 | 13.706 | 0.002 |
| $CO_2$ x Filtered | 1 | 1.53E-06 | 1.53E-06 | 0.324 | 0.577 |
| *Ulva* x Filtered | 1 | 2.17E-09 | 2.17E-09 | 0.000462 | 0.983 |
| $CO_2$ x *Ulva* x Filtered | 1 | 9.2E-06 | 9.2E-06 | 1.953 | 0.181 |
| Residual | 16 | 7.53E-05 | 4.71E-06 | | |
| Total | 23 | 0.000375 | 1.63E-05 | | |

Three-way analysis of variance of the tissue nitrogen for *Gracilaria* for the August experiment

| Source of Variation | DF | SS | MS | F | P |
|---|---|---|---|---|---|
| $CO_2$ | 1 | 2.43E-08 | 2.43E-08 | 0.00305 | 0.957 |
| *Ulva* | 1 | 4.39E-05 | 0.0000439 | 5.512 | 0.032 |
| Filtered | 1 | 2.11E-05 | 0.0000211 | 2.648 | 0.123 |
| $CO_2$ x *Ulva* | 1 | 3.68E-06 | 3.68E-06 | 0.461 | 0.507 |
| $CO_2$ x Filtered | 1 | 4.6E-06 | 0.0000046 | 0.577 | 0.459 |
| *Ulva* x Filtered | 1 | 4.29E-09 | 4.29E-09 | 0.000538 | 0.982 |
| $CO_2$ x *Ulva* x Filtered | 1 | 7.11E-06 | 7.11E-06 | 0.892 | 0.359 |
| Residual | 16 | 0.000128 | 7.97E-06 | | |
| Total | 23 | 0.000208 | 9.05E-06 | | |

Three-way analysis of variance of the tissue nitrogen for *Gracilaria* for the September experiment

| Source of Variation | DF | SS | MS | F | P |
|---|---|---|---|---|---|
| $CO_2$ | 1 | 0.000022 | 0.000022 | 6.983 | 0.014 |
| *Ulva* | 1 | 3.59E-05 | 3.59E-05 | 11.416 | 0.002 |
| Filtered | 1 | 0.000101 | 0.000101 | 31.959 | <0.001 |
| $CO_2$ x *Ulva* | 1 | 8.3E-06 | 8.3E-06 | 2.635 | 0.118 |
| $CO_2$ x Filtered | 1 | 0.000018 | 0.000018 | 5.714 | 0.025 |
| *Ulva* x Filtered | 1 | 1.08E-07 | 1.08E-07 | 0.0344 | 0.854 |
| $CO_2$ x *Ulva* x Filtered | 1 | 7.43E-08 | 7.43E-08 | 0.0236 | 0.879 |
| Residual | 24 | 7.56E-05 | 3.15E-06 | | |
| Total | 31 | 0.000261 | 8.41E-06 | | |

Three-way analysis of variance of the tissue nitrogen for *Gracilaria* for the October experiment

| Source of Variation | DF | SS | MS | F | P |
|---|---|---|---|---|---|
| $CO_2$ | 1 | 1.17E-05 | 0.0000117 | 1.283 | 0.269 |
| *Ulva* | 1 | 0.000131 | 0.000131 | 14.353 | <0.001 |
| Filtered | 1 | 0.000091 | 0.000091 | 9.985 | 0.004 |
| $CO_2$ x *Ulva* | 1 | 1.87E-05 | 0.0000187 | 2.046 | 0.166 |
| $CO_2$ x Filtered | 1 | 2.13E-06 | 2.13E-06 | 0.233 | 0.634 |
| *Ulva* x Filtered | 1 | 2.17E-05 | 0.0000217 | 2.38 | 0.137 |
| $CO_2$ x *Ulva* x Filtered | 1 | 7.12E-06 | 7.12E-06 | 0.781 | 0.386 |
| Residual | 23 | 0.00021 | 9.11E-06 | | |
| Total | 30 | 0.000479 | 0.000016 | | |

Three-way analysis of variance of the tissue C:N for *Gracilaria* for the early July experiment

| Source of Variation | DF | SS | MS | F | P |
|---|---|---|---|---|---|
| $CO_2$ | 1 | 57.836 | 57.836 | 3.625 | 0.075 |
| *Ulva* | 1 | 17.293 | 17.293 | 1.084 | 0.313 |
| Filtered | 1 | 533.096 | 533.096 | 33.41 | <0.001 |
| $CO_2$ x *Ulva* | 1 | 17.141 | 17.141 | 1.074 | 0.315 |
| $CO_2$ x Filtered | 1 | 68.366 | 68.366 | 4.285 | 0.055 |
| *Ulva* x Filtered | 1 | 97.61 | 97.61 | 6.117 | 0.025 |
| $CO_2$ x *Ulva* x Filtered | 1 | 47.512 | 47.512 | 2.978 | 0.104 |
| Residual | 16 | 255.296 | 15.956 | | |
| Total | 23 | 1094.15 | 47.572 | | |

Three-way analysis of variance of the tissue C:N for *Gracilaria* for the late July experiment

| Source of Variation | DF | SS | MS | F | P |
|---|---|---|---|---|---|
| $CO_2$ | 1 | 22.778 | 22.778 | 2.254 | 0.153 |
| *Ulva* | 1 | 32.128 | 32.128 | 3.179 | 0.094 |
| Filtered | 1 | 441.833 | 441.833 | 43.718 | <0.001 |
| $CO_2$ x *Ulva* | 1 | 68.615 | 68.615 | 6.789 | 0.019 |
| $CO_2$ x Filtered | 1 | 18.677 | 18.677 | 1.848 | 0.193 |
| *Ulva* x Filtered | 1 | 0.0995 | 0.0995 | 0.00985 | 0.922 |
| $CO_2$ x *Ulva* x Filtered | 1 | 0.019 | 0.019 | 0.00188 | 0.966 |
| Residual | 16 | 161.705 | 10.107 | | |
| Total | 23 | 745.855 | 32.428 | | |

Three-way analysis of variance of the tissue C:N for *Gracilaria* for the August experiment

| Source of Variation | DF | SS | MS | F | P |
|---|---|---|---|---|---|
| $CO_2$ | 1 | 3.495 | 3.495 | 0.356 | 0.559 |
| *Ulva* | 1 | 52.291 | 52.291 | 5.334 | 0.035 |
| Filtered | 1 | 6.656 | 6.656 | 0.679 | 0.422 |
| $CO_2$ x *Ulva* | 1 | 6.612 | 6.612 | 0.674 | 0.424 |
| $CO_2$ x Filtered | 1 | 3.627 | 3.627 | 0.37 | 0.552 |
| *Ulva* x Filtered | 1 | 1.194 | 1.194 | 0.122 | 0.732 |
| $CO_2$ x *Ulva* x Filtered | 1 | 13.916 | 13.916 | 1.42 | 0.251 |
| Residual | 16 | 156.855 | 9.803 | | |
| Total | 23 | 244.644 | 10.637 | | |

Three-way analysis of variance of the tissue C:N for *Gracilaria* for the September experiment

| Source of Variation | DF | SS | MS | F | P |
|---|---|---|---|---|---|
| $CO_2$ | 1 | 66.139 | 66.139 | 12.563 | 0.002 |
| *Ulva* | 1 | 28.729 | 28.729 | 5.457 | 0.029 |
| Filtered | 1 | 74.106 | 74.106 | 14.077 | 0.001 |
| $CO_2$ x *Ulva* | 1 | 0.578 | 0.578 | 0.11 | 0.743 |
| $CO_2$ x Filtered | 1 | 11.299 | 11.299 | 2.146 | 0.156 |
| *Ulva* x Filtered | 1 | 0.126 | 0.126 | 0.0239 | 0.878 |
| $CO_2$ x *Ulva* x Filtered | 1 | 3.086 | 3.086 | 0.586 | 0.452 |
| Residual | 23 | 121.084 | 5.265 | | |
| Total | 30 | 312.539 | 10.418 | | |

Three-way analysis of variance of the tissue C:N for *Gracilaria* for the October experiment

| Source of Variation | DF | SS | MS | F | P |
|---|---|---|---|---|---|
| $CO_2$ | 1 | 31.069 | 31.069 | 5.109 | 0.034 |
| *Ulva* | 1 | 104.004 | 104.004 | 17.104 | <0.001 |
| Filtered | 1 | 28.785 | 28.785 | 4.734 | 0.04 |
| $CO_2$ x *Ulva* | 1 | 24.465 | 24.465 | 4.023 | 0.057 |
| $CO_2$ x Filtered | 1 | 0.0994 | 0.0994 | 0.0163 | 0.899 |
| *Ulva* x Filtered | 1 | 7.524 | 7.524 | 1.237 | 0.277 |
| $CO_2$ x *Ulva* x Filtered | 1 | 0.677 | 0.677 | 0.111 | 0.742 |
| Residual | 23 | 139.857 | 6.081 | | |
| Total | 30 | 334.766 | 11.159 | | |

Three-way analysis of variance of the tissue carbon for *Ulva* for the early July experiment

| Source of Variation | DF | SS | MS | F | P |
|---|---|---|---|---|---|
| $CO_2$ | 1 | 0.000155 | 0.000155 | 0.903 | 0.356 |
| *Gracilaria* | 1 | 2.99E-05 | 2.99E-05 | 0.174 | 0.682 |
| Filtered | 1 | 0.000084 | 0.000084 | 0.49 | 0.494 |
| $CO_2$ x *Gracilaria* | 1 | 0.000683 | 0.000683 | 3.984 | 0.063 |
| $CO_2$ x Filtered | 1 | 0.000011 | 0.000011 | 0.0639 | 0.804 |
| *Gracilaria* x Filtered | 1 | 0.00131 | 0.00131 | 7.617 | 0.014 |
| $CO_2$ x *Gracilaria* x Filtered | 1 | 0.00053 | 0.00053 | 3.088 | 0.098 |
| Residual | 16 | 0.00274 | 0.000172 | | |
| Total | 23 | 0.00554 | 0.000241 | | |

Three-way analysis of variance of the tissue carbon for *Ulva* for the late July experiment

| Source of Variation | DF | SS | MS | F | P |
|---|---|---|---|---|---|
| $CO_2$ | 1 | 0.000687 | 0.000687 | 0.584 | 0.456 |
| *Gracilaria* | 1 | 8.2E-08 | 8.2E-08 | 6.97E-05 | 0.993 |
| Filtered | 1 | 0.00111 | 0.00111 | 0.942 | 0.346 |
| $CO_2$ x *Gracilaria* | 1 | 0.000543 | 0.000543 | 0.462 | 0.506 |
| $CO_2$ x Filtered | 1 | 0.00162 | 0.00162 | 1.377 | 0.258 |
| *Gracilaria* x Filtered | 1 | 0.0023 | 0.0023 | 1.955 | 0.181 |
| $CO_2$ x *Gracilaria* x Filtered | 1 | 0.000757 | 0.000757 | 0.644 | 0.434 |
| Residual | 16 | 0.0188 | 0.00118 | | |
| Total | 23 | 0.0258 | 0.00112 | | |

Three-way analysis of variance of the tissue carbon for *Ulva* for the August experiment

| Source of Variation | DF | SS | MS | F | P |
|---|---|---|---|---|---|
| $CO_2$ | 1 | 0.000125 | 0.000125 | 0.41 | 0.531 |
| *Gracilaria* | 1 | 0.000295 | 0.000295 | 0.965 | 0.341 |
| Filtered | 1 | 0.000138 | 0.000138 | 0.451 | 0.512 |
| $CO_2$ x *Gracilaria* | 1 | 9.52E-05 | 9.52E-05 | 0.312 | 0.584 |
| $CO_2$ x Filtered | 1 | 0.000059 | 0.000059 | 0.193 | 0.666 |
| *Gracilaria* x Filtered | 1 | 5.53E-05 | 5.53E-05 | 0.181 | 0.676 |
| $CO_2$ x *Gracilaria* x Filtered | 1 | 6.23E-08 | 6.23E-08 | 0.000204 | 0.989 |
| Residual | 16 | 0.00488 | 0.000305 | | |
| Total | 23 | 0.00565 | 0.000246 | | |

Three-way analysis of variance of the tissue carbon for *Ulva* for the September experiment

| Source of Variation | DF | SS | MS | F | P |
|---|---|---|---|---|---|
| $CO_2$ | 1 | 0.000678 | 0.000678 | 1.555 | 0.225 |
| *Gracilaria* | 1 | 0.00049 | 0.00049 | 1.124 | 0.3 |
| Filtered | 1 | 8.16E-05 | 8.16E-05 | 0.187 | 0.669 |
| $CO_2$ x *Gracilaria* | 1 | 0.00114 | 0.00114 | 2.614 | 0.12 |
| $CO_2$ x Filtered | 1 | 0.00454 | 0.00454 | 10.412 | 0.004 |
| *Gracilaria* x Filtered | 1 | 0.000552 | 0.000552 | 1.267 | 0.272 |
| $CO_2$ x *Gracilaria* x Filtered | 1 | 4E-08 | 4E-08 | 9.18E-05 | 0.992 |
| Residual | 23 | 0.01 | 0.000436 | | |
| Total | 30 | 0.0178 | 0.000594 | | |

Three-way analysis of variance of the tissue carbon for *Ulva* for the October experiment

| Source of Variation | DF | SS | MS | F | P |
|---|---|---|---|---|---|
| $CO_2$ | 1 | 0.00176 | 0.00176 | 2.109 | 0.159 |
| *Gracilaria* | 1 | 1.65E-05 | 1.65E-05 | 0.0198 | 0.889 |
| Filtered | 1 | 4.8E-07 | 4.8E-07 | 0.000575 | 0.981 |
| $CO_2$ x *Gracilaria* | 1 | 0.000157 | 0.000157 | 0.188 | 0.668 |
| $CO_2$ x Filtered | 1 | 0.000204 | 0.000204 | 0.244 | 0.626 |
| *Gracilaria* x Filtered | 1 | 0.000031 | 0.000031 | 0.0372 | 0.849 |
| $CO_2$ x *Gracilaria* x Filtered | 1 | 0.000433 | 0.000433 | 0.518 | 0.479 |
| Residual | 24 | 0.02 | 0.000835 | | |
| Total | 31 | 0.0226 | 0.00073 | | |

Three-way analysis of variance of the tissue nitrogen for *Ulva* for the early July experiment

| Source of Variation | DF | SS | MS | F | P |
|---|---|---|---|---|---|
| $CO_2$ | 1 | 3.17E-06 | 3.17E-06 | 1.414 | 0.252 |
| *Gracilaria* | 1 | 5.26E-06 | 5.26E-06 | 2.343 | 0.145 |
| Filtered | 1 | 0.000191 | 0.000191 | 85.053 | <0.001 |
| $CO_2$ x *Gracilaria* | 1 | 1.69E-06 | 1.69E-06 | 0.751 | 0.399 |
| $CO_2$ x Filtered | 1 | 6.17E-08 | 6.17E-08 | 0.0275 | 0.87 |
| *Gracilaria* x Filtered | 1 | 1.18E-05 | 1.18E-05 | 5.254 | 0.036 |
| $CO_2$ x *Gracilaria* x Filtered | 1 | 7.32E-07 | 7.32E-07 | 0.326 | 0.576 |
| Residual | 16 | 3.59E-05 | 2.25E-06 | | |
| Total | 23 | 0.00025 | 1.09E-05 | | |

Three-way analysis of variance of the tissue nitrogen for *Ulva* for the late July experiment

| Source of Variation | DF | SS | MS | F | P |
|---|---|---|---|---|---|
| $CO_2$ | 1 | 2.38E-06 | 2.38E-06 | 0.216 | 0.648 |
| *Gracilaria* | 1 | 8.07E-07 | 8.07E-07 | 0.0733 | 0.79 |
| Filtered | 1 | 6.64E-05 | 0.0000664 | 6.021 | 0.026 |
| $CO_2$ x *Gracilaria* | 1 | 1.64E-06 | 1.64E-06 | 0.148 | 0.705 |
| $CO_2$ x Filtered | 1 | 3.5E-07 | 3.5E-07 | 0.0318 | 0.861 |
| *Gracilaria* x Filtered | 1 | 4.31E-06 | 4.31E-06 | 0.391 | 0.54 |
| $CO_2$ x *Gracilaria* x Filtered | 1 | 2.33E-06 | 2.33E-06 | 0.211 | 0.652 |
| Residual | 16 | 0.000176 | 0.000011 | | |
| Total | 23 | 0.000254 | 0.0000111 | | |

Three-way analysis of variance of the tissue nitrogen for *Ulva* for the August experiment

| Source of Variation | DF | SS | MS | F | P |
|---|---|---|---|---|---|
| $CO_2$ | 1 | 5.27E-06 | 5.27E-06 | 0.658 | 0.429 |
| *Gracilaria* | 1 | 1.67E-05 | 0.0000167 | 2.091 | 0.167 |
| Filtered | 1 | 8.51E-05 | 0.0000851 | 10.634 | 0.005 |
| $CO_2$ x *Gracilaria* | 1 | 2.8E-06 | 0.0000028 | 0.349 | 0.563 |
| $CO_2$ x Filtered | 1 | 2.79E-06 | 2.79E-06 | 0.349 | 0.563 |
| *Gracilaria* x Filtered | 1 | 5.27E-05 | 0.0000527 | 6.585 | 0.021 |
| $CO_2$ x *Gracilaria* x Filtered | 1 | 3.42E-06 | 3.42E-06 | 0.427 | 0.523 |
| Residual | 16 | 0.000128 | 8.01E-06 | | |
| Total | 23 | 0.000297 | 0.0000129 | | |

Three-way analysis of variance of the tissue nitrogen for *Ulva* for the September experiment

| Source of Variation | DF | SS | MS | F | P |
|---|---|---|---|---|---|
| $CO_2$ | 1 | 2.9E-06 | 0.0000029 | 1.488 | 0.235 |
| *Gracilaria* | 1 | 1.88E-06 | 1.88E-06 | 0.963 | 0.337 |
| Filtered | 1 | 2.13E-05 | 0.0000213 | 10.912 | 0.003 |
| $CO_2$ x *Gracilaria* | 1 | 1.93E-06 | 1.93E-06 | 0.991 | 0.33 |
| $CO_2$ x Filtered | 1 | 5.78E-09 | 5.78E-09 | 0.00296 | 0.957 |
| *Gracilaria* x Filtered | 1 | 1.18E-05 | 0.0000118 | 6.049 | 0.022 |
| $CO_2$ x *Gracilaria* x Filtered | 1 | 2.61E-06 | 2.61E-06 | 1.341 | 0.259 |
| Residual | 23 | 4.48E-05 | 1.95E-06 | | |
| Total | 30 | 0.000089 | 2.97E-06 | | |

Three-way analysis of variance of the tissue nitrogen for *Ulva* for the October experiment

| Source of Variation | DF | SS | MS | F | P |
|---|---|---|---|---|---|
| $CO_2$ | 1 | 3.18E-09 | 3.18E-09 | 0.001 | 0.975 |
| *Gracilaria* | 1 | 5.93E-06 | 5.93E-06 | 1.867 | 0.184 |
| Filtered | 1 | 3.06E-06 | 3.06E-06 | 0.964 | 0.336 |
| $CO_2$ x *Gracilaria* | 1 | 7.32E-07 | 7.32E-07 | 0.231 | 0.635 |
| $CO_2$ x Filtered | 1 | 8.15E-08 | 8.15E-08 | 0.0257 | 0.874 |
| *Gracilaria* x Filtered | 1 | 0.0000015 | 0.0000015 | 0.474 | 0.498 |
| $CO_2$ x *Gracilaria* x Filtered | 1 | 2.06E-07 | 2.06E-07 | 0.065 | 0.801 |
| Residual | 24 | 0.0000762 | 3.17E-06 | | |
| Total | 31 | 0.0000877 | 2.83E-06 | | |

Three-way analysis of variance of the tissue C:N for *Ulva* for the early July experiment

| Source of Variation | DF | SS | MS | F | P |
|---|---|---|---|---|---|
| $CO_2$ | 1 | 60.304 | 60.304 | 3.115 | 0.097 |
| *Gracilaria* | 1 | 5.327 | 5.327 | 0.275 | 0.607 |
| Filtered | 1 | 2024.355 | 2024.355 | 104.559 | <0.001 |
| $CO_2$ x *Gracilaria* | 1 | 3.167 | 3.167 | 0.164 | 0.691 |
| $CO_2$ x Filtered | 1 | 22.906 | 22.906 | 1.183 | 0.293 |
| *Gracilaria* x Filtered | 1 | 28.628 | 28.628 | 1.479 | 0.242 |
| $CO_2$ x *Gracilaria* x Filtered | 1 | 6.405 | 6.405 | 0.331 | 0.573 |
| Residual | 16 | 309.776 | 19.361 | | |
| Total | 23 | 2460.867 | 106.994 | | |

Three-way analysis of variance of the tissue C:N for *Ulva* for the late July experiment

| Source of Variation | DF | SS | MS | F | P |
|---|---|---|---|---|---|
| $CO_2$ | 1 | 6.605 | 6.605 | 0.192 | 0.667 |
| *Gracilaria* | 1 | 14.493 | 14.493 | 0.422 | 0.525 |
| Filtered | 1 | 661.367 | 661.367 | 19.261 | <0.001 |
| $CO_2$ x *Gracilaria* | 1 | 27.963 | 27.963 | 0.814 | 0.38 |
| $CO_2$ x Filtered | 1 | 11.549 | 11.549 | 0.336 | 0.57 |
| *Gracilaria* x Filtered | 1 | 0.00146 | 0.00146 | 4.24E-05 | 0.995 |
| $CO_2$ x *Gracilaria* x Filtered | 1 | 6.484 | 6.484 | 0.189 | 0.67 |
| Residual | 16 | 549.389 | 34.337 | | |
| Total | 23 | 1277.85 | 55.559 | | |

Three-way analysis of variance of the tissue C:N for *Ulva* for the August experiment

| Source of Variation | DF | SS | MS | F | P |
|---|---|---|---|---|---|
| $CO_2$ | 1 | 21.361 | 21.361 | 1.211 | 0.287 |
| *Gracilaria* | 1 | 8.219 | 8.219 | 0.466 | 0.505 |
| Filtered | 1 | 305.054 | 305.054 | 17.289 | <0.001 |
| $CO_2$ x *Gracilaria* | 1 | 18.795 | 18.795 | 1.065 | 0.317 |
| $CO_2$ x Filtered | 1 | 15.42 | 15.42 | 0.874 | 0.364 |
| *Gracilaria* x Filtered | 1 | 146.02 | 146.02 | 8.276 | 0.011 |
| $CO_2$ x *Gracilaria* x Filtered | 1 | 23.864 | 23.864 | 1.352 | 0.262 |
| Residual | 16 | 282.314 | 17.645 | | |
| Total | 23 | 821.046 | 35.698 | | |

Three-way analysis of variance of the tissue C:N for *Ulva* for the September experiment

| Source of Variation | DF | SS | MS | F | P |
|---|---|---|---|---|---|
| $CO_2$ | 1 | 55.191 | 55.191 | 3.585 | 0.071 |
| *Gracilaria* | 1 | 0.675 | 0.675 | 0.0439 | 0.836 |
| Filtered | 1 | 272.751 | 272.751 | 17.717 | <0.001 |
| $CO_2$ x *Gracilaria* | 1 | 1.711 | 1.711 | 0.111 | 0.742 |
| $CO_2$ x Filtered | 1 | 27.663 | 27.663 | 1.797 | 0.193 |
| *Gracilaria* x Filtered | 1 | 61.005 | 61.005 | 3.963 | 0.059 |
| $CO_2$ x *Gracilaria* x Filtered | 1 | 17.035 | 17.035 | 1.107 | 0.304 |
| Residual | 23 | 354.08 | 15.395 | | |
| Total | 30 | 808.275 | 26.943 | | |

Three-way analysis of variance of the tissue C:N for *Ulva* for the October experiment

| Source of Variation | DF | SS | MS | F | P |
|---|---|---|---|---|---|
| $CO_2$ | 1 | 26.543 | 26.543 | 1.646 | 0.212 |
| *Gracilaria* | 1 | 73.064 | 73.064 | 4.531 | 0.044 |
| Filtered | 1 | 34.41 | 34.41 | 2.134 | 0.157 |
| $CO_2$ x *Gracilaria* | 1 | 22.149 | 22.149 | 1.373 | 0.253 |
| $CO_2$ x Filtered | 1 | 0.101 | 0.101 | 0.00626 | 0.938 |
| *Gracilaria* x Filtered | 1 | 13.702 | 13.702 | 0.85 | 0.366 |
| $CO_2$ x *Gracilaria* x Filtered | 1 | 0.45 | 0.45 | 0.0279 | 0.869 |
| Residual | 24 | 387.028 | 16.126 | | |
| Total | 31 | 557.447 | 17.982 | | |

**Supplementary Tables S3**

Tissue δ$^{13}$C content (‰) of dry tissue samples of *Gracilaria* and *Ulva* for July through October experiments. Values represent means ± standard deviation.

***Gracilaria***

| | Treatment | Early July | Late July | August | September | October |
|---|---|---|---|---|---|---|
| Filtered | Control | -12.76±1.67 | -12.46±0.77 | -12.51±0.90 | -12.90±0.48 | -12.05±0.30 |
| | Ulva | -13.41±0.22 | -12.65±0.81 | -15.19±2.14 | -13.15±1.47 | -12.63±1.21 |
| | $CO_2$ | -34.66±2.99 | -21.12±1.70 | -19.34±1.28 | -18.60±0.24 | -22.80±0.92 |
| | $CO_2$/Ulva | -28.79±0.85 | -26.18±2.02 | -18.33±1.15 | -18.77±1.29 | -24.01±0.92 |
| Unfiltered | Control | -14.40±1.41 | -14.45±1.13 | -12.28±0.42 | -13.00±1.38 | -13.07±1.78 |
| | Ulva | -15.43±0.63 | -14.40±2.24 | -11.90±0.62 | -13.35±0.95 | -13.30±0.56 |
| | $CO_2$ | -31.85±3.77 | -25.84±2.99 | -19.49±1.31 | -19.20±0.79 | -25.02±1.77 |
| | $CO_2$/Ulva | -33.84±2.47 | -27.22±8.94 | -21.73±1.63 | -18.67±0.55 | -22.17±2.72 |

***Ulva***

| | Treatment | Early July | Late July | August | September | October |
|---|---|---|---|---|---|---|
| Filtered | Control | -11.88±2.35 | -8.58±2.46 | -8.10±0.64 | -12.61±3.30 | -12.90±4.50 |
| | Gracilaria | -8.48±1.12 | -9.57±2.43 | -9.92±4.35 | -12.51±0.90 | -15.19±2.14 |
| | $CO_2$ | -40.09±0.73 | -39.42±2.82 | -26.32±1.73 | -29.81±0.95 | -36.94±1.44 |
| | $CO_2$/Gracilaria | -27.63±5.59 | -38.32±4.60 | -27.37±3.95 | -31.51±0.86 | -32.92±5.28 |
| Unfiltered | Control | -11.38±1.31 | -13.91±2.73 | -12.89±1.77 | -14.76±2.70 | -12.43±5.41 |
| | Gracilaria | -9.33±2.71 | -12.92±1.25 | -11.16±2.45 | -16.50±0.91 | -14.23±3.87 |
| | $CO_2$ | -31.39±7.33 | -35.84±4.75 | -30.32±2.49 | -30.79±0.88 | -34.84±4.08 |
| | $CO_2$/Gracilaria | -35.59±4.44 | -38.73±4.85 | -31.57±2.43 | -31.91±1.81 | -38.21±1.26 |

**Supplementary Tables S4**

Tissue nitrogen content (g N per g dry tissue), tissue carbon content (g N per g dry tissue), and tissue C:N of dry tissue samples of *Gracilaria* and *Ulva* for July through October experiments. Values represent means ± standard deviation.

***Gracilaria***

*Tissue C content*

| | Treatment | Early July | Late July | August | September | October |
|---|---|---|---|---|---|---|
| Filtered | Control | 0.292±0.030 | 0.294±0.026 | 0.278±0.016 | 0.295±0.026 | 0.309±0.015 |
| | Ulva | 0.274±0.017 | 0.329±0.030 | 0.298±0.012 | 0.287±0.032 | 0.330±0.008 |
| | $CO_2$ | 0.309±0.018 | 0.289±0.012 | 0.300±0.026 | 0.308±0.028 | 0.326±0.012 |
| | $CO_2$/Ulva | 0.277±0.021 | 0.292±0.011 | 0.318±0.041 | 0.318±0.036 | 0.314±0.008 |
| Unfiltered | Control | 0.277±0.015 | 0.328±0.010 | 0.292±0.032 | 0.295±0.029 | 0.307±0.004 |
| | Ulva | 0.295±0.022 | 0.325±0.010 | 0.286±0.018 | 0.296±0.025 | 0.288±0.065 |
| | $CO_2$ | 0.274±0.024 | 0.331±0.011 | 0.302±0.016 | 0.310±0.042 | 0.308±0.008 |
| | $CO_2$/Ulva | 0.289±0.021 | 0.302±0.001 | 0.294±0.013 | 0.312±0.021 | 0.325±0.013 |

*Tissue N content*

| | Treatment | Early July | Late July | August | September | October |
|---|---|---|---|---|---|---|
| Filtered | Control | 0.021±0.004 | 0.019±0.001 | 0.022±0.004 | 0.024±0.001 | 0.025±0.004 |
| | Ulva | 0.016±0.004 | 0.022±0.002 | 0.017±0.004 | 0.021±0.001 | 0.022±0.002 |
| | $CO_2$ | 0.022±0.005 | 0.023±0.002 | 0.021±0.005 | 0.023±0.002 | 0.026±0.005 |
| | $CO_2$/Ulva | 0.018±0.002 | 0.017±0.002 | 0.020±0.001 | 0.022±0.001 | 0.017±0.002 |
| Unfiltered | Control | 0.011±0.001 | 0.015±0.002 | 0.019±0.003 | 0.022±0.002 | 0.020±0.002 |
| | Ulva | 0.020±0.008 | 0.016±0.001 | 0.017±0.001 | 0.019±0.003 | 0.018±0.004 |
| | $CO_2$ | 0.010±0.001 | 0.015±0.004 | 0.019±0.001 | 0.018±0.002 | 0.020±0.001 |
| | $CO_2$/Ulva | 0.011±0.002 | 0.012±0.001 | 0.016±0.002 | 0.017±0.001 | 0.017±0.003 |

*Tissue C:N*

| | | Early July | Late July | August | September | October |
|---|---|---|---|---|---|---|
| Filtered | Control | 16.4±2.1 | 18.1±3.1 | 15.3±2.4 | 14.3±1.3 | 14.7±2.2 |
| | Ulva | 19.9±2.9 | 17.2±0.4 | 21.3±5.7 | 16.0±1.4 | 17.9±1.6 |
| | $CO_2$ | 17.3±4.6 | 15.0±1.4 | 17.9±5.8 | 15.6±1.1 | 15.1±2.5 |
| | $CO_2$/Ulva | 18.5±0.5 | 20.7±2.0 | 18.7±1.5 | 17.1±2.0 | 21.3±2.5 |
| Unfiltered | Control | 29.3±3.3 | 25.1±3.2 | 17.6±0.9 | 15.6±1.6 | 17.8±1.9 |
| | Ulva | 19.1±7.7 | 23.9±0.8 | 19.6±0.1 | 18.4±1.7 | 18.4±3.2 |
| | $CO_2$ | 31.3±1.8 | 25.4±0.4 | 18.6±1.4 | 20.7±4.9 | 17.8±0.9 |
| | $CO_2$/Ulva | 30.1±4.6 | 31.0±7.4 | 21.6±1.3 | 21.6±2.4 | 22.6±3.8 |

***Ulva***

*Tissue C content*

| | Treatment | Early July | Late July | August | September | October |
|---|---|---|---|---|---|---|
| Filtered | Control | 0.302±0.004 | 0.259±0.073 | 0.296±0.008 | 0.280±0.015 | 0.315±0.036 |
| | Gracilaria | 0.315±0.008 | 0.300±0.044 | 0.288±0.022 | 0.251±0.023 | 0.307±0.019 |
| | $CO_2$ | 0.307±0.008 | 0.307±0.003 | 0.285±0.014 | 0.301±0.014 | 0.294±0.018 |
| | $CO_2$/Gracilaria | 0.318±0.019 | 0.306±0.014 | 0.285±0.028 | 0.297±0.018 | 0.309±0.023 |
| Unfiltered | Control | 0.302±0.015 | 0.320±0.033 | 0.292±0.005 | 0.299±0.033 | 0.315±0.046 |
| | Gracilaria | 0.305±0.009 | 0.299±0.008 | 0.278±0.016 | 0.287±0.017 | 0.318±0.022 |
| | $CO_2$ | 0.328±0.006 | 0.313±0.027 | 0.286±0.014 | 0.272±0.019 | 0.298±0.037 |
| | $CO_2$/Gracilaria | 0.291±0.024 | 0.295±0.013 | 0.280±0.022 | 0.285±0.020 | 0.295±0.014 |

*Tissue N content*

| | Treatment | Early July | Late July | August | September | October |
|---|---|---|---|---|---|---|
| Filtered | Control | 0.013±0.001 | 0.013±0.006 | 0.017±0.004 | 0.012±0.003 | 0.011±0.003 |
| | Gracilaria | 0.015±0.003 | 0.014±0.005 | 0.012±0.001 | 0.009±0.001 | 0.010±0.002 |
| | $CO_2$ | 0.012±0.001 | 0.012±0.001 | 0.017±0.006 | 0.010±0.001 | 0.011±0.001 |
| | $CO_2$/Gracilaria | 0.014±0.001 | 0.013±0.001 | 0.012±0.001 | 0.010±0.001 | 0.010±0.001 |
| Unfiltered | Control | 0.008±0.001 | 0.011±0.004 | 0.010±0.001 | 0.009±0.001 | 0.010±0.003 |
| | Gracilaria | 0.009±0.002 | 0.009±0.001 | 0.013±0.003 | 0.009±0.001 | 0.009±0.001 |
| | $CO_2$ | 0.008±0.001 | 0.009±0.001 | 0.010±0.001 | 0.008±0.001 | 0.011±0.002 |
| | $CO_2$/Gracilaria | 0.007±0.001 | 0.010±0.001 | 0.010±0.001 | 0.009±0.001 | 0.009±0.001 |

*Tissue C:N*

| | | Early July | Late July | August | September | October |
|---|---|---|---|---|---|---|
| Filtered | Control | 27.7±0.6 | 26.7±8.8 | 21.0±4.8 | 28.3±6.8 | 35.6±5.1 |
| | Gracilaria | 24.9±4.9 | 26.3±5.2 | 27.3±1.3 | 31.8±4.9 | 35.5±4.7 |
| | $CO_2$ | 29.2±1.7 | 30.3±2.9 | 21.5±7.2 | 33.8±2.8 | 32.0±4.1 |
| | $CO_2$/Gracilaria | 25.8±1.3 | 27.6±3.2 | 27.4±0.9 | 35.4±1.2 | 35.6±4.7 |
| Unfiltered | Control | 42.9±3.5 | 37.6±9.3 | 33.5±3.2 | 40.4±2.2 | 36.3±3.7 |
| | Gracilaria | 42.4±9.5 | 39.2±4.0 | 25.9±6.9 | 35.3±2.2 | 39.2±2.1 |
| | $CO_2$ | 46.3±1.0 | 40.4±5.1 | 33.2±0.7 | 39.2±3.7 | 32.9±4.5 |
| | $CO_2$/Gracilaria | 49.3±4.7 | 35.7±4.6 | 33.2±2.4 | 38.1±4.0 | 38.7±1.9 |
